# Bioactive Conjugate Based on Bacterial Protein Toxins for Targeted Down-Modulation of Macrophage Functions

**DOI:** 10.3390/ijms27188433

**Published:** 2026-09-21

**Authors:** Johanna Diehm, Marie Jachmann, Joscha Borho, Robin Schönegg, Anna Elbe, Marc Spitzmüller, Michael Fauler, Manfred Frick, Holger Barth

**Affiliations:** 1 Institute of Experimental and Clinical Pharmacology, Toxicology and Pharmacology of Natural Products, University of Ulm Medical Center, 89081 Ulm, Germany; johanna.diehm@uni-ulm.de (J.D.); marie.jachmann@uni-ulm.de (M.J.); joscha.borho@alumni.uni-ulm.de (J.B.); 2 Institute for General Physiology, University of Ulm, 89081 Ulm, Germany; robin.schoenegg@uni-ulm.de (R.S.); anna.elbe@uni-ulm.de (A.E.); marc.spitzmueller@uni-ulm.de (M.S.); michael.fauler@uni-ulm.de (M.F.)

**Keywords:** CyaA toxin, bacterial protein toxins, C3lim, targeted delivery, monocytes, macrophages, down-modulation, cAMP, migration, chemotaxis

## Abstract

Targeted down-modulation of monocytes and macrophages is a promising strategy for controlling excessive inflammatory processes. Bacterial protein toxins are attractive pharmacological modulation tools for this purpose because of their potency and specificity. Here, we combined the specific properties of the two protein toxins C3lim from *Clostridium limosum* and CyaA from *Bordetella pertussis* in the preferentially monocyte/macrophage-targeting bioconjugate C2IN-C3lim_E174Q_-Cys_His-AC. The enzymatically inactive and monocytic cell-targeting transporter C2IN-C3lim_E174Q_-Cys was recombinantly produced and chemically coupled to the isolated adenylate cyclase (AC) domain of CyaA, which mediates down-modulation of monocytic cell functions through intracellular cAMP accumulation Within 1 h, the bioconjugate showed preferential cell association with monocytic cells relative to HeLa cells, which served as the non-target cell model, without impairing overall cellular viability. Intracellular enzymatic activity was successfully confirmed by significantly elevated cAMP levels in all target cell types. Moreover, in migration assays with primary human monocytes, the bioconjugate markedly reduced both general motility and targeted chemotaxis, demonstrating that the delivery of the enzyme moiety translates into a measurable functional response. Therefore, we conclude that the novel bioconjugate C2IN-C3lim_E174Q_-Cys_His-AC enables preferential targeting of monocytes and macrophages under the experimental conditions investigated here and provides proof-of-concept for a targeted protein delivery platform for functional modulation of monocytic cells.

## 1. Introduction

Monocytic cells are a crucial part of the innate human immune system, but they are also involved in the development and progression of severe diseases [1]. In particular, the excessive activation of macrophages can drive inflammatory processes in organs and tissues, which can result in life-threatening conditions including systemic inflammation in the context of severe illness like physical trauma and post-traumatic complications [1,2]. Some relevant examples for diseases associated with excessive macrophage activity are the macrophage activation syndrome (MAS), rheumatoid arthritis, or pulmonary fibrosis [3,4,5]. Therefore, the targeted pharmacological down-modulation of macrophage activity as well as the differentiation of macrophages from monocytes has been considered a potential strategy for controlling excessive inflammatory responses [6,7].

One approach to selectively target monocytes and macrophages is the use of bacterial protein toxins, which are selectively taken up by these cells and modulate their functions [8]. We and others exploited the C3 toxin from *Clostridium* (*C*.) *botulinum* (C3bot) and *C. limosum* (C3lim) for this purpose because we found earlier that these C3 proteins are efficiently internalized into the cytosol of cells belonging to the myelomonocytic lineage including monocytes, macrophages, dendritic cells and osteoclasts but not into the cytosol of other cell types including epithelial cells, fibroblasts, osteoblasts and other blood cells [9,10]. Most likely, C3 toxins bind to integrin-like receptors on the surface of monocytic cells, which triggers their endocytic uptake, but the precise mechanism of their uptake into cells is not understood so far [11]. In the cytosol, the C3 toxins mono-ADP-ribosylate the monomeric GTP-binding proteins RhoA, -B and -C, thereby inhibiting the Rho-mediated signal transduction of the cells [8,12]. Since Rho is the master regulator of actin dynamics in cells, treatment of monocytic cells with C3 toxins results in down-modulation of their actin-mediated processes, including exocytosis, phagocytosis, and migration [8,13].

We reported earlier that the fusion of the C2IN protein to C3 toxin enhances its transport into the cytosol [14]. C2IN is the N-terminal domain of C2I, the enzyme component of the binary C2 toxin from *C. botulinum* and most likely, C2IN enhances the intracellular transport of C3 toxin from the endosomal lumen across endosomal membranes into the cytosol [14,15]. Furthermore, recombinant C2IN-C3lim has been shown to selectively target cells of the monocytic lineage, including macrophage-like cells and primary human monocytes, and to be efficiently internalized by osteoclast-like cells. In contrast, no comparable uptake was observed in non-monocytic pre-osteoblastic cells [14,15]. We previously used the recombinant C2IN-C3lim fusion toxin to demonstrate the inhibitory effect of C3 toxin on the excessive monocyte migration from the blood into the lungs after blunt chest trauma in vivo in mice [14]. Moreover, enzymatically inactive and non-toxic C3_E174Q_ serves for selective and safe delivery of cargo proteins and enzymes into the cytosol of monocytic cells [10]. Previous studies have demonstrated that C3bot_E174Q_ is taken up substantially more by primary human macrophages than by lymphocytes [10]. This finding supports the preferential targeting of cells of the monocytic lineage by C3bot_E174Q_ and highlights its potential as an attractive system for targeted drug delivery [10,16].

In addition to C3 toxins, the adenylyl cyclase toxin CyaA from *Bordetella* (*B*.) *pertussis* was used to down-modulate macrophage activity, as well as monocyte-to-macrophage transition [17,18]. Moreover, CyaA de-differentiates terminally differentiated human alveolar macrophages into monocyte-like cells [18]. CyaA binds to myeloid phagocytes via complement receptor 3 (CD11b/CD18) and delivers its adenylyl cyclase domain (AC) across the cytoplasmic membrane into the cytosol [19,20]. There, AC activity converts ATP into cAMP, which activates protein kinase A, thereby preventing the expression of macrophage markers [18]. Primary human monocytes treated with CyaA remain small and lack pseudopodia. Moreover, treatment of terminally differentiated macrophages, e.g., human alveolar macrophages, resulted in de-differentiation of these cells into monocyte-like cells, as measured by the loss of macrophage marker expression and the appearance of CD14 [17,18]. However, CyaA is taken up into other cell types including PMNs and is, therefore, less suitable for the selective pharmacological modulation of macrophages *in vivo* [19,21]. Therefore, the selective delivery of the isolated catalytic domain of CyaA into the cytosol of macrophages would be an important step towards a selective pharmacological approach to down-modulate excessive macrophage functions in the context of the abovementioned diseases.

Here, we generated a bioactive biomolecule by chemical coupling of the recombinantly produced proteins C2IN-C3lim_E174Q_ as a monocyte/macrophage-targeting transporter and AC, the isolated adenylate cyclase domain of CyaA, as an enzyme moiety for the down-modulation of cell functions. Preferential targeting of monocytes and macrophages under the experimental conditions investigated here was supported by our findings, and the effects of the bioconjugate on cellular functions were investigated.

## 2. Results

### 2.1. Generation of the Monocyte-Selective Bioconjugate C2IN-C3lim_E174Q_-Cys_His-AC

**Figure 1:**
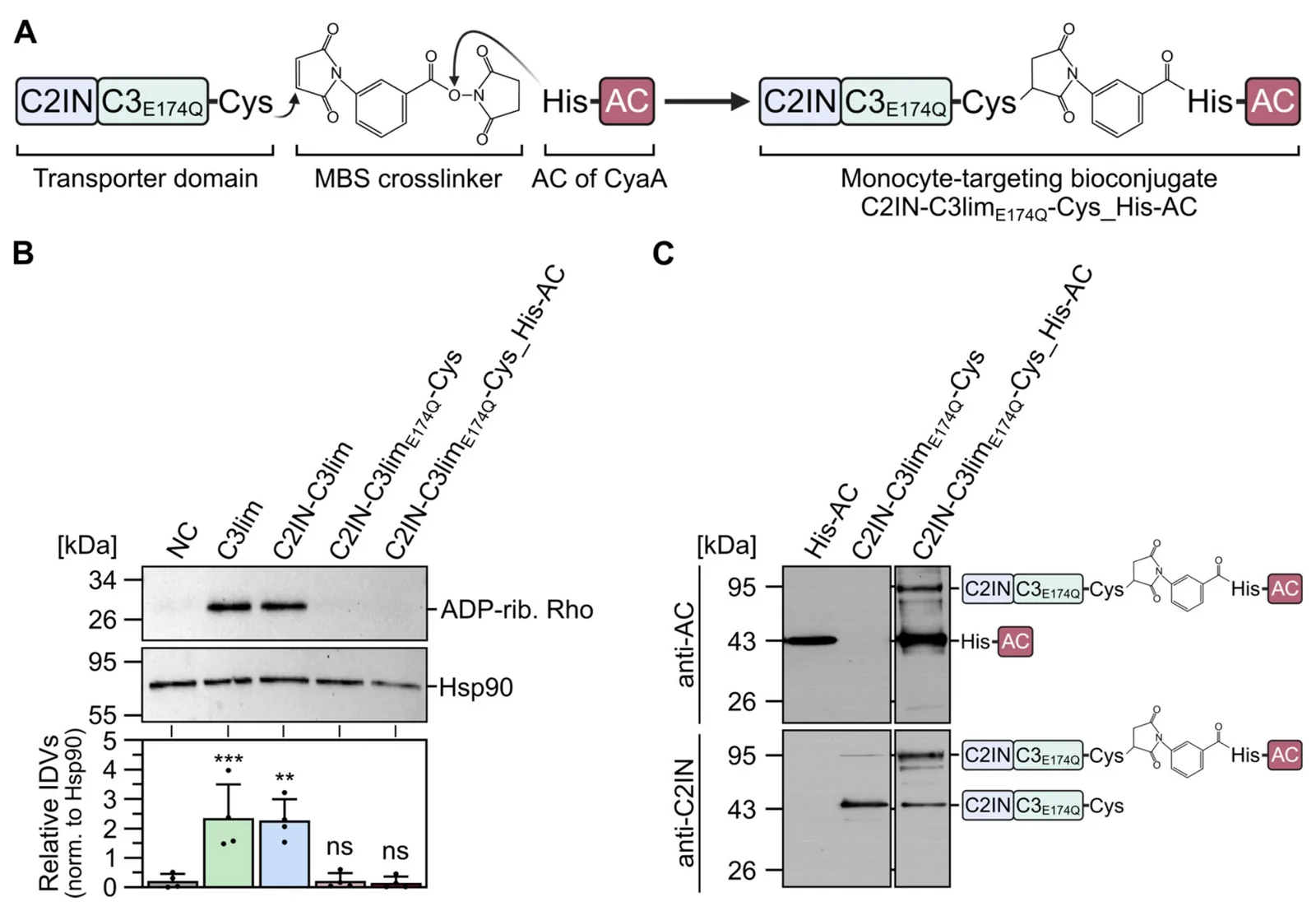
Generation of the monocyte-targeting bioconjugate C2IN-C3lim_E174Q_-Cys_His-AC. (**A**) Schematic representation of the chemical crosslinking of the C3-toxin-derived monocyte-targeting transporter domain C2IN-C3lim_E174Q_-Cys with the enzymatically active adenylate cyclase (AC) domain of the CyaA toxin using an MBS crosslinker. (**B**) Proof of enzymatic inactivity of the transporter domain. *Upper panel:* Western blot of HeLa cell lysate incubated with C3lim toxin, C2IN-C3lim fusion toxin, C2IN-C3lim_E174Q_-Cys (including an inactivating point mutation) or the complete bioconjugate C2IN-C3lim_E174Q_-Cys_His-AC. The amount of ADP-ribosylated Rho is shown. *Lower panel:* Quantification of the Western blot with normalization of ADP-ribosylated Rho to total protein amount shown as relative integrated density values (IDVs). Four individual experiments were analyzed (*n* = 4). The assumptions of normality and homogeneity of variances were considered met based on the Shapiro–Wilk and Brown–Forsythe tests, respectively (all *p* > 0.05), followed by ordinary one-way ANOVA with Dunnett’s multiple comparisons test against the NC group. (ns, not significant; ** *p* < 0.005; *** *p* < 0.0005). (**C**) Western blot of His-AC, C2IN-C3lim_E174Q_-Cys, and C2IN-C3lim_E174Q_-Cys_His-AC after crosslinking. Antibodies directed against AC (upper panel) or C2IN (lower panel) are used.

To generate a monocyte- and macrophage-targeting delivery system for AC, recombinantly expressed and affinity-purified histidine-tagged AC (His-AC) and C2IN-C3lim_E174Q_ containing a C-terminal cysteine residue (C2IN-C3lim_E174Q_-Cys) were chemically coupled using the heterobifunctional crosslinker *m*-maleimidobenzoyl-*N*-hydroxysuccinimide ester (MBS). Specifically, MBS was first reacted with primary amine groups of His-AC, which introduced maleimide groups, followed by covalent coupling to the free thiol group of the engineered C-terminal cysteine residue of C2IN-C3lim_E174Q_-Cys, resulting in the formation of a stable thioether-linked bioconjugate (Figure 1A). Successful formation of the C2IN-C3lim_E174Q_-Cys_His-AC bioconjugate was verified by Western blot analysis. Following SDS-PAGE and immunoblotting against both AC and C2IN, a distinct higher molecular mass band corresponding to the expected combined mass of the conjugate (~94 kDa) was detected. Since the signals obtained with anti-AC and anti-C2IN antibodies showed complete co-detection, the presence of both protein moieties within the C2IN-C3lim_E174Q_-Cys_His-AC bioconjugate was confirmed (Figure 1C). To further optimize bioconjugate formation, the stoichiometric ratios of the coupling partners were determined. Coupling efficiency was analyzed by SDS-PAGE under non-reducing conditions by varying the molar ratios of His-AC to MBS and MBS-activated His-AC to C2IN-C3lim_E174Q-_Cys. Under these conditions, an His-AC:MBS ratio of 1:5 and an equimolar ratio of MBS-His-AC and C2IN-C3lim_E174Q_-Cys yielded the highest amount of desired bioconjugate with the lowest proportion of unconjugated protein (Supplementary Figure S1A). These optimized conjugation conditions were, therefore, employed for all subsequent analyses. Furthermore, coupling efficiency was evaluated across three independent coupling reactions at 31.3 ± 7.8% (mean ± SD, *n* = 3; Supplementary Figure S1B,C). To assess bioconjugate integrity, C2IN-C3lim_E174Q_-Cys_His-AC was analyzed over time under storage and cell culture conditions (Supplementary Figure S2). Approximately 93% and 50% of the initial bioconjugate signal remained after incubation in medium containing 5% and 10% heat-inactivated FBS for 24 h, respectively (Supplementary Figure S2C). These findings indicate that the bioconjugate retains sufficient integrity over the experimentally relevant 24 h period to support its use under the applied cell culture conditions.

As C2IN-C3lim_E174Q_-Cys is intended to serve as a targeted delivery platform for AC in monocytes and macrophages, the transporter must be devoid of intrinsic C3 enzyme activity. In particular, the conjugate must lack ADP-ribosyltransferase activity to ensure its use as an enzymatically inert transport moiety. We previously demonstrated that mutation of the catalytic glutamate residue to glutamine (E174Q) completely abolishes the Rho ADP-ribosyltransferase activity of C3bot [10]. Given the high sequence homology between C3bot and C3lim, an analogous loss of enzymatic activity is expected for C3lim_E174Q_. To verify the enzymatic inactivity of the transporter construct, the ADP-ribosylation activity of C2IN-C3lim_E174Q_-Cys and the corresponding C2IN-C3lim_E174Q_-Cys_His-AC bioconjugate was assessed by in vitro ADP-ribosylation assay (Figure 1B). In agreement with the predicted effect of the point mutation E174Q, neither the transporter nor the bioconjugate exhibited ADP-ribosylation of Rho, demonstrating the complete absence of C3 activity. These findings establish C2IN-C3lim_E174Q_-Cys as an enzymatically inert delivery platform and confirm that the generated bioconjugate can be employed to investigate AC-mediated effects in monocytic cells.

### 2.2. The Bioconjugate C2IN-C3lim_E174Q_-Cys_His-AC Preferentially Targets Monocytic Cells

**Figure 2:**
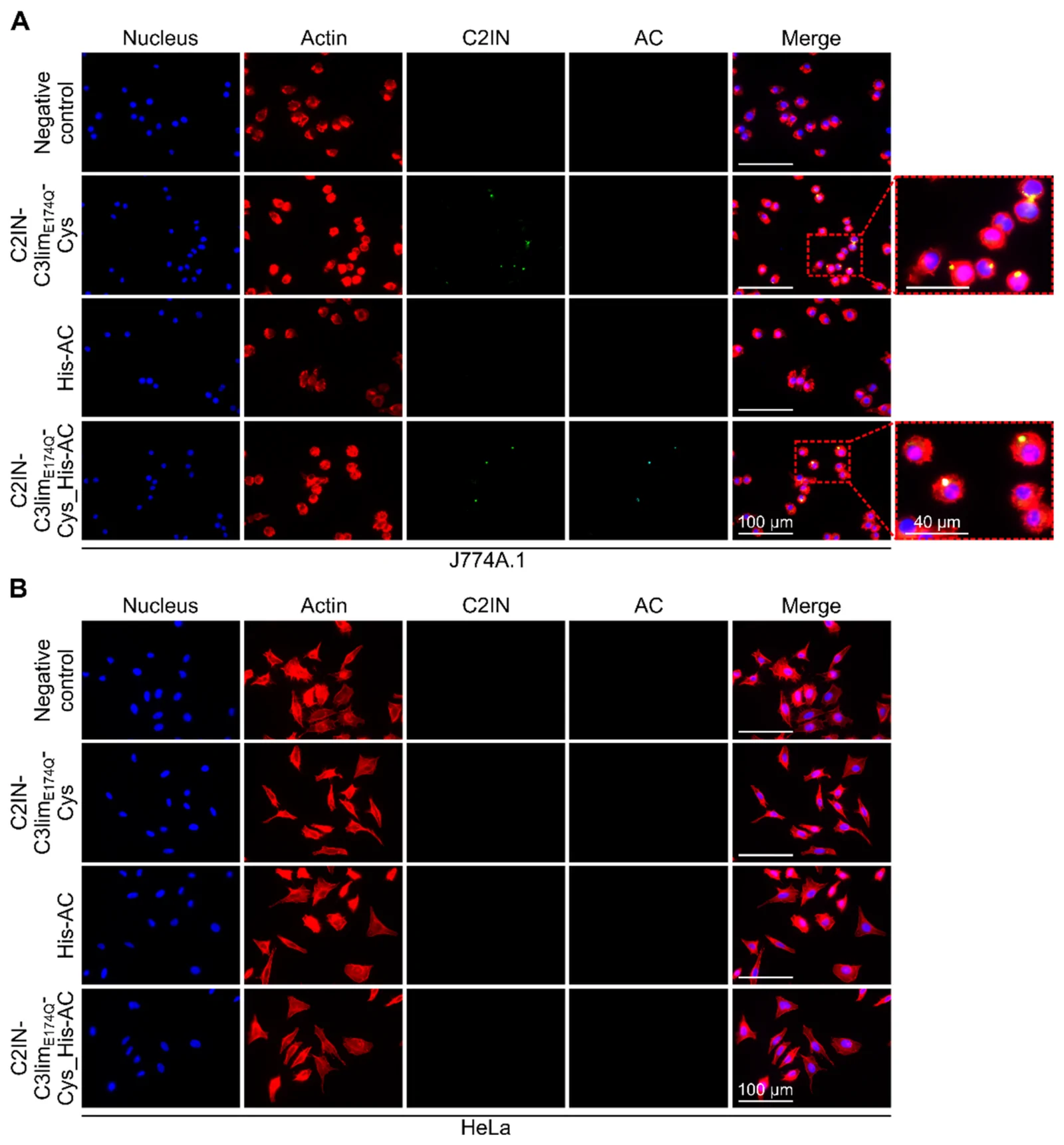
Preferential targeting by C2IN-C3lim_E174Q_-Cys_His-AC relative to HeLa cells. (**A**) J774A.1 cells were treated with 160 nM C2IN-C3lim_E174Q_-Cys, His-AC or C2IN-C3lim_E174Q_-Cys_His-AC. After 1 h, cells were washed and fixed, and immunostaining was performed using SiR-Actin (red, actin) and Hoechst (blue, nucleus). Cell association of C2IN-C3lim_E174Q_-Cys, His-AC or C2IN-C3lim_E174Q_-Cys_His-AC was assessed by fluorescence microscopy using antibodies directed against C2IN (green) and AC (cyan). (**B**) Representative pictures of fluorescence microscopic analysis of HeLa cells treated as described in (**A**). Scale bars are shown in representative images and correspond to 100 µm for all micrographs and 40 µm for all digital magnifications.

To evaluate the transporter-mediated delivery of AC into target cells by the bioconjugate, immunofluorescence microscopy was employed. Therefore, murine macrophages (J774A.1 cells) were incubated for 1 h with either C2IN-C3lim_E174Q_-Cys, His-AC, or the bioconjugate C2IN-C3lim_E174Q_-Cys_His-AC. The cellular association of the respective proteins was analyzed by immunofluorescence staining using antibodies against AC and C2IN (Figure 2A). Visible colocalization of both the AC and C2IN signals demonstrates that both bioconjugate moieties are simultaneously associated with the target cells, thus supporting C2IN-C3lim_E174Q_-Cys-mediated targeting of AC to macrophages. Furthermore, the transporter alone was detected in association with J774A.1 cells, demonstrating its ability to interact with target cells independently. In contrast, no cell-associated signal was observed for His-AC alone, indicating inefficient targeting of the cells by the enzyme moiety in the absence of the transporter moiety.

To assess the preferential targeting of the transporter-mediated delivery approach relative to a non-target cell model, the cellular association of the bioconjugate with non-target Hela cells was investigated. Therefore, epithelial HeLa cells were incubated with the bioconjugate under the same experimental conditions used for J774A.1 cells, and the cellular localization of C2IN-C3lim_E174Q_-Cys, His-AC, or the bioconjugate C2IN-C3lim_E174Q_-Cys_His-AC was assessed by fluorescence microscopy (Figure 2B). In contrast to the pronounced cell-associated signal in J774A.1 macrophages, no detectable intracellular fluorescence signal was observed in HeLa cells. The absence of AC and C2IN signals indicates that the neither the transporter alone nor the bioconjugate efficiently associates with non-target HeLa cells, supporting preferential targeting of monocytic cells over HeLa cells under the experimental conditions investigated here.

**Figure 3:**
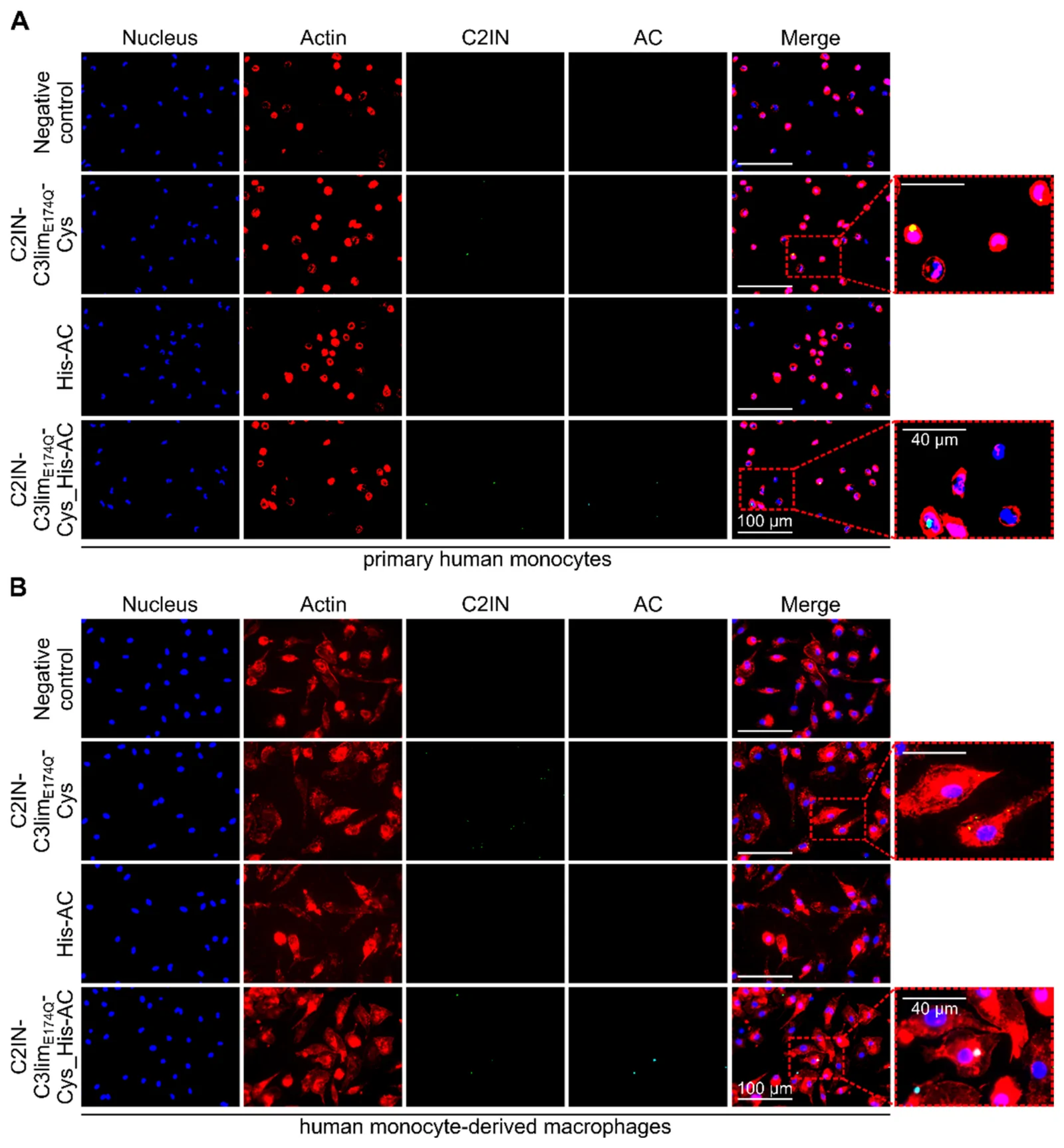
Targeting of primary human monocytes and monocyte-derived macrophages by C2IN-C3lim_E174Q_-Cys_His-AC. (**A**) Primary human monocytes isolated from human buffy coats were treated 20 h after isolation with 160 nM C2IN-C3lim_E174Q_-Cys, His-AC or C2IN-C3lim_E174Q_-Cys_His-AC for 1 h. Cells were then washed and fixed, and immunostaining was performed using SiR-Actin (red, actin) and Hoechst (blue, nucleus). Cellular localization of C2IN-C3lim_E174Q_-Cys, His-AC or C2IN-C3lim_E174Q_-Cys_His-AC was assessed by fluorescence microscopy using antibodies directed against C2IN (green) and AC (cyan). (**B**) Representative fluorescence microscopy images of monocyte-derived macrophages. Monocytes were isolated from human buffy coats and differentiated for 5 days in the presence of M-CSF. Treatment was performed on day 6 as described in (**A**). Scale bars are shown in representative images and correspond to 100 µm for all micrographs and 40 µm for all digital magnifications.

Primary human monocytes and monocyte-derived macrophages (MDMs) as physiological target cells were investigated next to show the translational applicability of the bioconjugate. For this purpose, CD14^+^ monocytes were isolated from human blood and either used directly or differentiated into MDMs by stimulation with M-CSF, as previously described. The cellular localization of C2IN-C3lim_E174Q_-Cys, His-AC, or the bioconjugate C2IN-C3lim_E174Q_-Cys_His-AC into these cell types was assessed by fluorescence microscopy (Figure 3A,B). After 1 h of incubation, fluorescence signal corresponding to the transporter as well as the bioconjugate could be detected in association with both primary human monocytes and MDMs, indicating efficient targeting of primary human monocytic cells.

**Figure 4:**
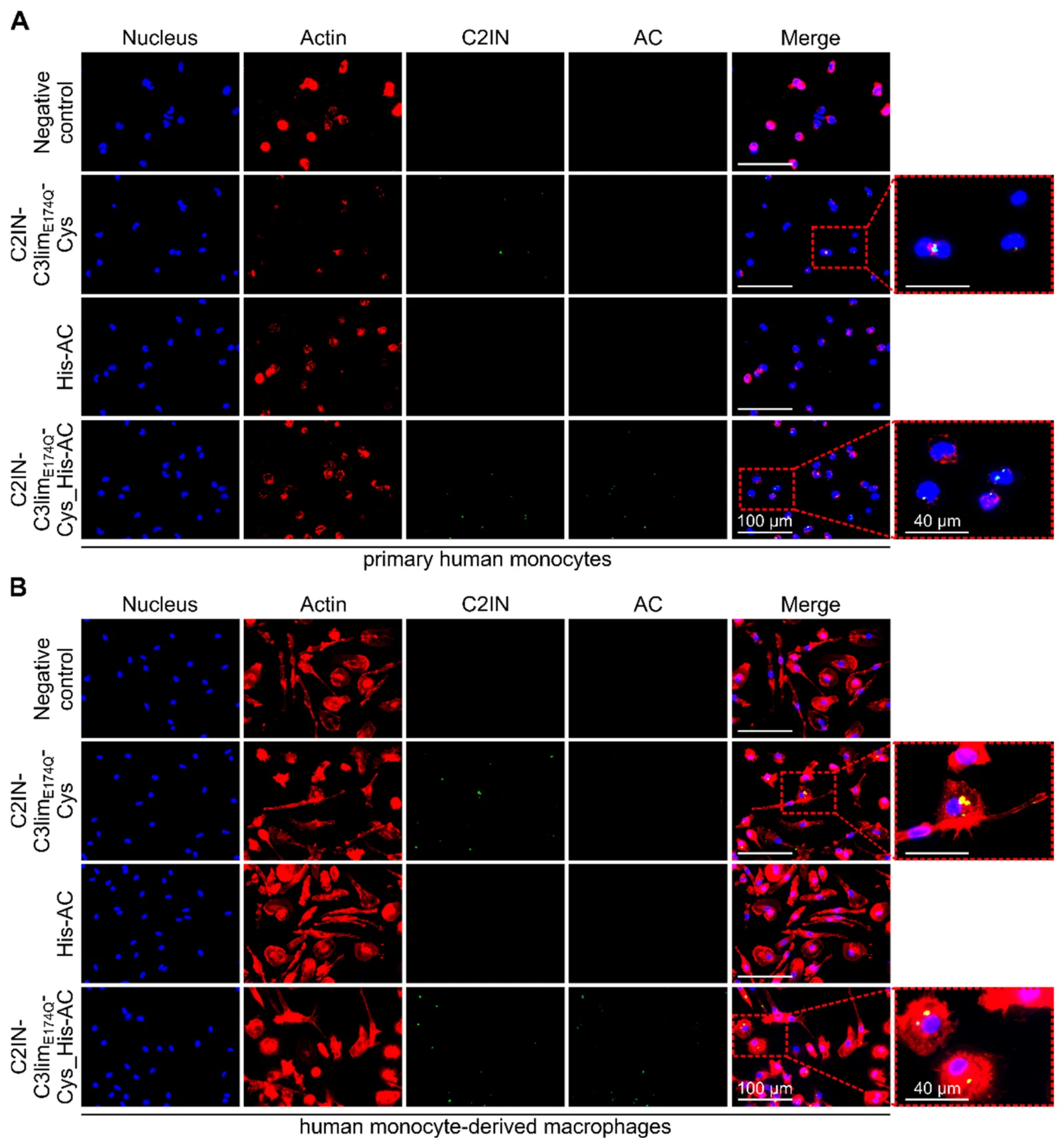
Time-dependent increase in cell-associated C2IN-C3lim_E174Q_-Cys_His-AC in primary human monocytes and monocyte-derived macrophages. (**A**) Primary human monocytes were isolated from buffy coats and exposed 20 h post-isolation to 160 nM C2IN-C3lim_E174Q_-Cys, His-AC, or C2IN-C3lim_E174Q_-Cys_His-AC for 18 h. Subsequently, cells were washed, fixed, and stained with SiR-Actin (red) and Hoechst (blue). Cellular targeting by C2IN-C3lim_E174Q_-Cys, His-AC or C2IN-C3lim_E174Q_-Cys_His-AC was analyzed by fluorescence microscopy using antibodies against C2IN (green) and AC (cyan). (**B**) Representative fluorescence microscopy images of monocyte-derived macrophages treated as described in (**A**). Scale bars are shown in representative images and correspond to 100 µm for all micrographs and 40 µm for all digital magnifications.

Analysis of time-dependent cellular detection revealed an increase in the number of bioconjugate-positive cells with sustained incubation. While cell-associated signals were detectable at early time points, prolonged exposure to C2IN-C3lim_E174Q_-Cys_His-AC resulted in a progressive increase in bioconjugate-associated fluorescence, with colocalized signals of C2IN and AC becoming prominent after 4 h and 18 h of incubation (Figure 4A,B and Figure S3). Although the extent of cellular association varied among individual cells, the overall trend demonstrated a time-dependent targeting of human monocytes and MDMs by the bioconjugate.

### 2.3. The Bioconjugate C2IN-C3lim_E174Q_-Cys_His-AC Does Not Affect the Viability of Target and Non-Target Cells

**Figure 5:**
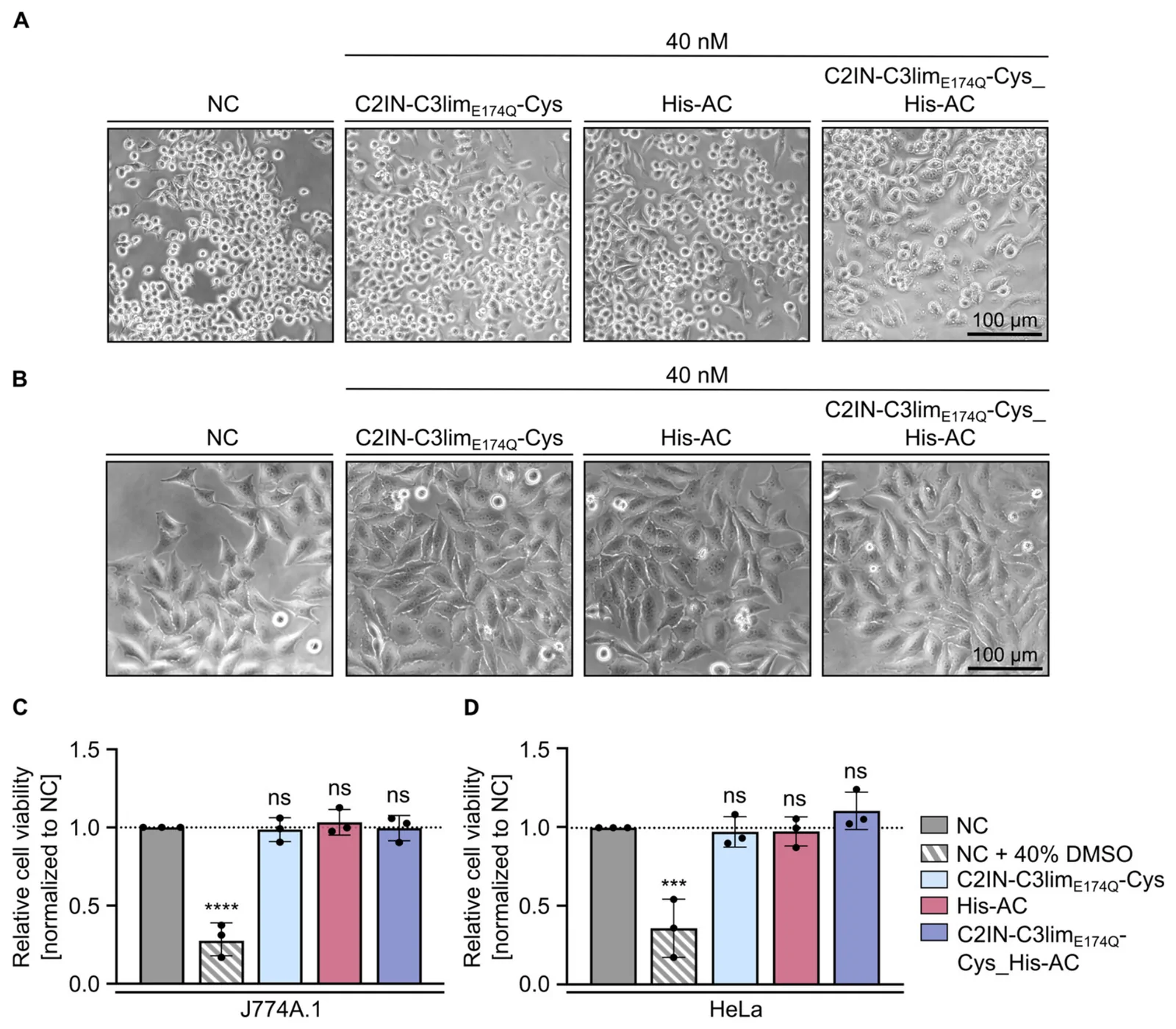
C2IN-C3lim_E174Q_-Cys_His-AC shows no cytotoxicity and differential effects on morphology in J774A.1 and HeLa cells. (**A**,**B**) Representative bright field images of (**A**) J774A.1 cells or (**B**) HeLa cells incubated for 48 h with 40 nM C2IN-C3lim_E174Q_-Cys_His-AC or respective controls (untreated cells, C2IN-C3lim_E174Q_-Cys, His-AC) are shown. (**C**,**D**) Cell viability quantification of (**C**) J774A.1 cells or (**D**) HeLa cells using the MTS assay after exposition for 48 h, as described in (**A**). As a positive control, the respective cells were incubated with 40% DMSO for 1 h before measurement. Values are normalized for every timepoint to the negative control (NC) and given as mean ± SD (*n* = 3) of triplicates from three independent experiments. Normality was assumed based on the Shapiro–Wilk test (all *p* > 0.05) and homogeneity of variances by the Brown–Forsythe test (all *p* > 0.05), followed by ordinary one-way ANOVA with Dunnett’s multiple comparisons test against the NC group. (ns, not significant, *** *p* < 0.0005, **** *p* < 0.0001). Scale bars are shown in representative images and correspond to 100 µm for all micrographs.

Following the observation of efficient targeting of monocytic cells by the bioconjugate, its effects on cellular morphology and metabolic activity were investigated as indicators of cellular viability. Therefore, J774A.1 target cells, as well as HeLa cells, used as a non-target cell model, were incubated with either C2IN-C3lim_E174Q_-Cys, His-AC or the bioconjugate C2IN-C3lim_E174Q_-Cys_His-AC for 48 h. Cellular morphology was subsequently assessed by bright-field microscopy, while metabolic activity was evaluated in a terminal endpoint analysis using an MTS assay as a surrogate parameter for cellular viability. J774A.1 cells treated with C2IN-C3lim_E174Q_-Cys_His-AC showed subtle morphological alterations visible as a tendency towards a more pronounced cell spreading, accompanied by the appearance of intracellular inclusions (Figure 5A). Exposure to the individual protein moieties did not result in comparable morphological changes. In contrast, non-target HeLa cells did not display apparent changes in cell morphology following bioconjugate treatment, indicating a differential cellular effect between the investigated target and non-target cell models (Figure 5B).

To further evaluate whether the observed morphological alterations were associated with impaired cellular function, metabolic activity was quantified by MTS assays. No significant differences in metabolic activity were detected between treated and non-treated conditions in either J774A.1 or HeLa cells (Figure 5C,D). These findings indicate that the morphological changes observed in target cells were not accompanied by a reduction in metabolic activity and, therefore, do not suggest an impairment of cellular viability upon exposure to the bioconjugate.

### 2.4. Cell Type-Selective Targeting by C2IN-C3lim_E174Q_-Cys_His-AC Results in Significantly Increased Intracellular cAMP

Since our findings suggested preferential targeting of monocytic cells relative to HeLa cells by the bioconjugate without considerable effects on cellular viability, the intracellular enzymatic activity of AC as the cargo moiety of C2IN-C3lim_E174Q_-Cys_His-AC was investigated. Once localized within the cytosol, AC of CyaA is activated by binding to endogenous calmodulin, thereby enabling the conversion of ATP to cAMP [21]. Consequently, intracellular cAMP levels were used as a functional readout of cytosolic delivery and enzymatic activity of the AC cargo moiety within the bioconjugate C2IN-C3lim_E174Q_-Cys_His-AC.

Next, we aimed to improve the purity of C2IN-C3lim_E174Q_-Cys_His-AC further by size-exclusion chromatography (SEC), resulting in a moderate increase in purity accompanied by protein loss (Supplementary Figure S4A–D). Functional assessment by cAMP ELISA revealed similar activity of SEC-purified and non-SEC-purified bioconjugate preparations, indicating that residual protein components did not substantially contribute to the observed functional activity (Supplementary Figure S4E). SEC was, therefore, omitted from subsequent experiments to allow for preservation of the protein yield while maintaining comparable functional activity.

**Figure 6:**
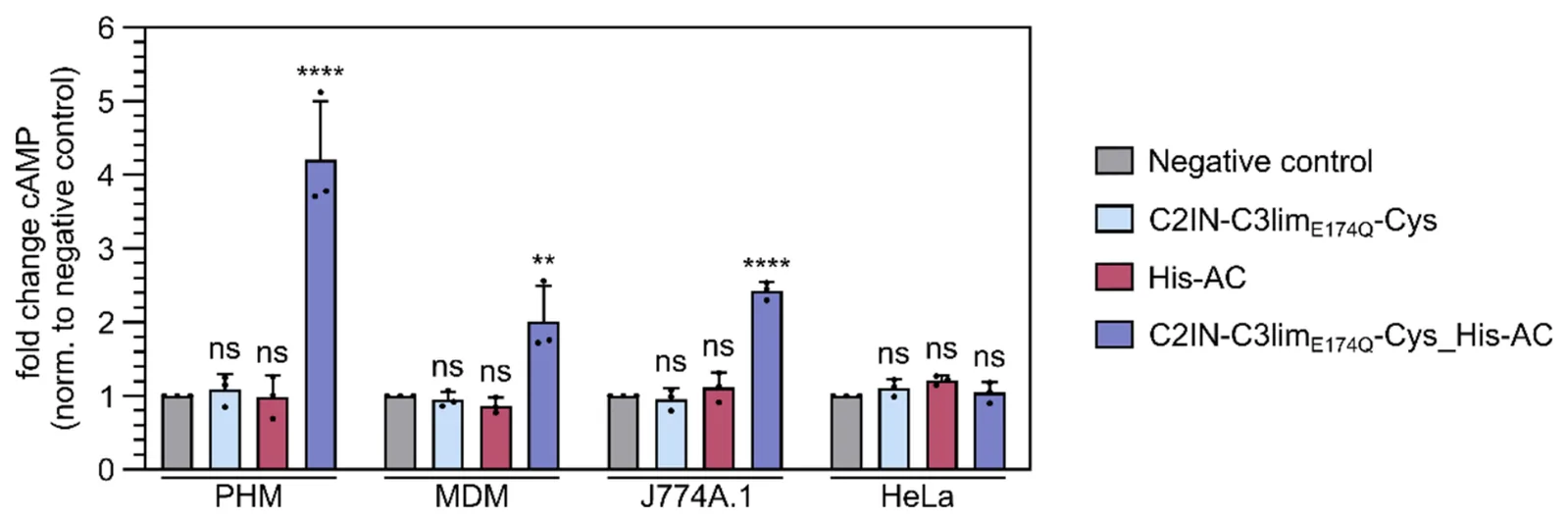
Preferential targeting of monocytic cells by C2IN-C3lim_E174Q_-Cys_His-AC results in significant increase in intracellular cAMP. Primary human monocytes (PHMs), monocyte-derived macrophages (MDMs), J774A.1 and HeLa cells were preincubated with the PDE4 inhibitor rolipram prior to stimulation with C2IN-C3lim_E174Q_-Cys, His-AC, or C2IN-C3lim_E174Q_-Cys_His-AC at cell type-specific concentrations (PHM: 80 nM; MDM: 120 nM; J774A.1: 40 nM; HeLa: 40 nM). After 1 h of treatment, cells were washed and intracellular cAMP levels were quantified using a cAMP ELISA. Data are presented as fold change relative to untreated controls. Values are given as mean ± SD (*n* = 3) of duplicates from three individual experiments. Normality was not rejected by the Shapiro–Wilk test (all *p* > 0.05), and homogeneity of variances was supported by the Brown–Forsythe test (all *p* > 0.05). Data were subsequently analyzed by ordinary one-way ANOVA with Dunnett’s multiple comparisons test against the NC group (ns, not significant; ** *p* < 0.005; **** *p* < 0.0001).

The intracellular activity of C2IN-C3lim_E174Q_-Cys_His-AC was subsequently investigated in distinct target cell models, including primary human CD14+ monocytes (PHM), monocyte-derived macrophages (MDMs), and the murine macrophage cell line J774A.1. In addition, HeLa cells were included as a non-target cell model. Following treatment with C2IN-C3lim_E174Q_-Cys, His-AC, or the bioconjugate C2IN-C3lim_E174Q_-Cys_His-AC for 1 h, cells were lysed and cAMP concentrations were quantified using a cAMP ELISA. Exposure with the bioconjugate resulted in a significant increase in cAMP levels in all investigated target cell types (Figure 6). Conversely, exposure of non-target HeLa cells to the bioconjugate did not induce changes in cAMP concentrations.

Collectively, these results demonstrate that C2IN-C3lim_E174Q_-Cys_His-AC not only enables preferential targeting of monocytic cells under the experimental conditions investigated here but also preserves its enzymatic activity. The increase in cAMP observed in target cells, together with the absence of a detectable effect in HeLa cells, further supports preferential targeting of monocytic cells relative to the HeLa non-target model and highlights its potential for targeted modulation of intracellular signaling pathways in monocytic cells.

### 2.5. C2IN-C3lim_E174Q_-Cys_His-AC Results in a Dose-Dependent Immobilization of Monocytes and Macrophages but Does Not Affect Migration Speed in Mobile Cells

**Figure 7:**
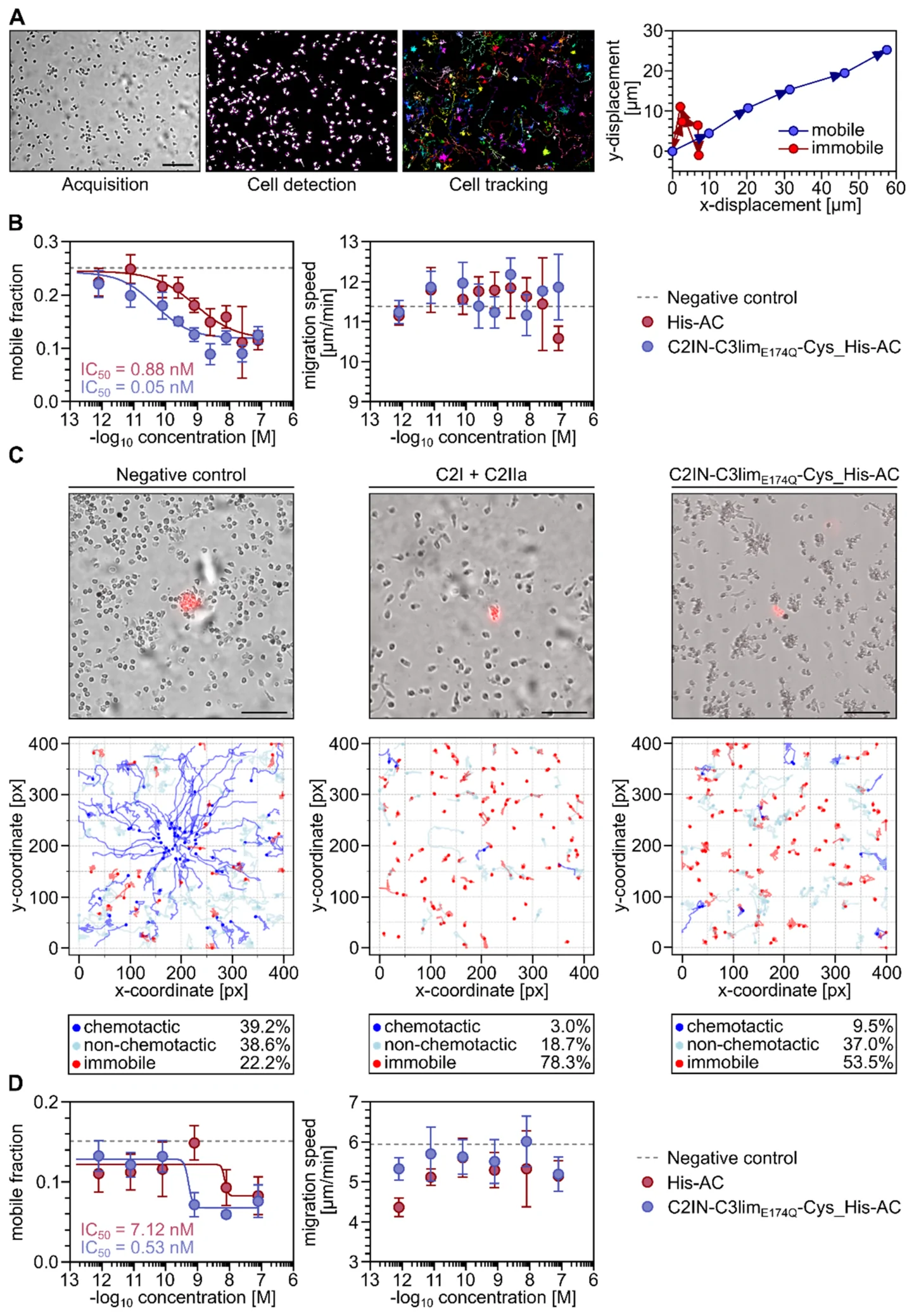
C2IN-C3lim_E174Q_-Cys_His-AC results in a dose-dependent immobilization of monocytes and macrophages but does not affect migration speed. (**A**) To analyze monocyte motility, the cells (left, brightfield image) were detected using the Laplacian-of-Gaussian detector (cell detection) and linked over consecutive frames using the Kalman tracker (cell tracking). *Right Graph:* Net displacement (the straight-line distance from the starting point to the endpoint, as indicated by the arrows in the graph) over five consecutive frames was used to distinguish between motile and immobile cells. The scale bar corresponds to 100 µm. (**B**) Analysis of the fraction of mobile monocytes (left) and the migration speed of mobile monocytes (right) treated with increasing doses of C2IN-C3lim_E174Q_-Cys_His-AC or His-AC. Control indicates the fraction of mobile cells and their speed in untreated monocytes. Data are represented as the mean ± SEM (*n* = 9–11 with *n* wells from 10 donors). (**C**) *Top row:* Brightfield images of macrophages and zymosan particles (red). The scale bar corresponds to 100 µm. *Bottom row*: Tracks of cells depicted in the top row of images. The trajectories were classified into three populations: chemotactic motility towards a zymosan particle (blue); non-chemotactic motility (turquoise); and immobility (red). The numbers indicate the percentage of each population. (**D**) Analysis of the fraction of chemotactic macrophages (left) and the migration speed of chemotactic macrophages (right) treated with increasing doses of C2IN-C3lim_E174Q_-Cys_His-AC or His-AC. Control indicates the fraction of mobile cells and their speed in untreated macrophages. Data are represented as the mean ± SEM (*n* = 3–6).

Next, we analyzed the impact of C2IN-C3lim_E174Q_-Cys_His-AC on the cellular motility of monocytic cells. In initial experiments we examined the effect of C2IN-C3lim_E174Q_-Cys_His-AC on the spontaneous motility of freshly isolated primary human monocytes. Primary human monocytes were incubated with different concentrations of either C2IN-C3lim_E174Q_-Cys_His-AC or His-AC or left untreated (control). The motility of cells was monitored over 5 h and the migration of single cells analyzed (Figure 7A). The results of these experiments revealed that C2IN-C3lim_E174Q_-Cys_His-AC reduced the fraction of mobile monocytes at subnanomolar concentrations and in a dose-dependent manner (IC_50_ ≈ 0.05 nM, 95% CI [0.015, 0.15]). Interestingly, the migration speed of mobile cells was not affected. Similar effects were observed with His-AC alone, but His-AC was less potent than C2IN-C3lim_E174Q_-Cys_His-AC (approx. 18 fold) (Figure 7B).

Subsequent experiments analyzed the effect of C2IN-C3lim_E174Q_-Cys_His-AC on the chemotaxis of macrophages that had been differentiated from freshly isolated primary human monocytes. After cell isolation, the cells were preincubated for 18 h with different concentrations of C2IN-C3lim_E174Q_-Cys_His-AC, His-AC, the binary *C. botulinum* C2 toxin (C2I/C2IIa), which was included as a positive control based on its well-established effects on actin-dependent cellular functions or left untreated (control) [22,23]. Chemotaxis towards zymosan particles was then analyzed over a 2 h period (Figure 7C). Consistent with its known biological activity, C2 toxin markedly reduced the fraction of chemotactic macrophages. As with the effects observed for monocyte motility, C2IN-C3lim_E174Q_-Cys_His-AC reduced the fraction of chemotactic macrophages in a dose-dependent manner at subnanomolar concentrations (IC_50_ ≈ 0.53 nM, 95% CI [0.47, 0.59]). However, it did not affect the migration speed of chemotactic cells. Again, His-AC induced similar effects, albeit with much lower potency (Figure 7D).

## 3. Discussion

Monocytes and macrophages contribute to pulmonary host defense, inflammatory regulation and tissue repair [1,6]. However, persistent activation and accumulation of these cells contribute substantially to the initiation and progression of inflammatory lung diseases, including acute respiratory distress syndrome (ARDS), pulmonary fibrosis and trauma-associated lung injury [2,5,24]. Owing to their pivotal role in orchestrating inflammatory responses, selective modulation of macrophage function has emerged as an attractive therapeutic strategy [1,7]. Nevertheless, the efficient and targeted intracellular delivery of biologically active proteins into these cells remains a major challenge [8,10].

The present study demonstrates that an enzymatically inactive C3 toxin-derived transporter chemically conjugated to an enzymatically active protein cargo can be used to preferentially target monocytes and macrophages under the conditions investigated here. The generated bioconjugate showed preferential targeting of murine macrophages, primary human monocytes, as well as monocyte-derived macrophages, and induced a significant increase in intracellular cAMP levels in these cell types, whereas no changes in intracellular cAMP have been detected in non-target HeLa cells, which served as the non-target cell model in the present study. Thus, the observed preferential targeting should be interpreted relative to HeLa cells rather than as evidence of absolute cell type selectivity. Importantly, intracellular delivery occurred without detectable impairment of cellular metabolic activity under the investigated conditions. Our findings establish proof-of-concept for intracellular delivery of a biologically active protein cargo into monocytic cells and demonstrate measurable functional modulation of target cells in vitro. Further studies in disease-relevant models will be required to determine whether these cellular effects can be translated into therapeutic benefit in vivo. The bioconjugate investigated in the present study was generated by chemical conjugation of the C3lim_E174Q_-derived transporter and the AC domain of the CyaA toxin. Although recombinant fusion proteins generally provide homogeneous products with defined stoichiometry, recombinant fusion of the C2IN-C3lim_E174Q_ transporter and the catalytically active AC domain would require generation of a novel chimeric toxin comprising functional domains from two different bacterial toxins. Such recombinant constructs may raise additional biosafety considerations during protein production and handling and would require the generation of a new recombinant construct whenever an alternative cargo is to be investigated. In contrast, the strategy presented here relies on the independent production and purification of both protein components followed by their assembly through chemical crosslinking. In addition to avoiding recombinant production of a novel hybrid toxin, independent production of transporter and cargo also provides considerable flexibility for future applications. Different cargo proteins could be coupled to the same transport system by chemical conjugation, allowing their intracellular delivery to be evaluated without generating a new recombinant fusion protein.

Full-length CyaA has evolved as a potent bacterial toxin that delivers its AC domain into host cells [25]. However, its biological activity is not restricted to intracellular cAMP production [26]. In addition to translocation of the AC domain, CyaA functions as a pore-forming RTX toxin and induces membrane permeabilization, resulting in AC-independent effects on target cells [21,26]. Moreover, cellular uptake is primarily mediated by complement receptor 3 (CR3, CD11b/CD18), which is highly expressed on myeloid cells but is not restricted to monocytes and macrophages [19,26]. Consequently, the biological responses elicited by full-length CyaA would reflect the combined activities of multiple toxin domains and cannot be attributed solely to intracellular cAMP signaling [26].

Catalytically active C3 toxins likewise represent a potential strategy for modulating monocyte and macrophage function [8]. Following selective cellular uptake, they irreversibly ADP-ribosylate small Rho GTPases, thereby disrupting Rho-dependent signaling pathways that regulate actin cytoskeletal organization, migration, phagocytosis and vesicular trafficking [8,12,13]. Although this mechanism efficiently suppresses macrophage function, the biological activity is inseparably linked to the intrinsic enzymatic function of the toxin, limiting its applicability as a versatile intracellular delivery platform.

The bioconjugate developed in the present study addresses these limitations by combining the isolated AC domain with an enzymatically inactive C3-derived transporter. This design preserves the preferential macrophage-targeting properties of C3 observed in the experimental system investigated here, while restricting its function to intracellular delivery, thereby eliminating Rho-dependent effects of the transporter itself. At the same time, use of the isolated AC domain avoids the additional toxin-associated activities of full-length CyaA, allowing for the observed cellular responses to be attributed predominantly to intracellular cAMP signaling. More generally, separating preferential cellular targeting from biological activity enables the biological response to be assigned clearly to the conjugated cargo rather than the transport protein itself, thereby providing a modular strategy for evaluating alternative intracellularly active proteins in macrophages.

Despite the encouraging proof-of-concept established in the present study, several aspects warrant further investigation. The MBS-mediated conjugation used here generates a heterogeneous reaction mixture containing both conjugated and unconjugated protein species. While the MBS-based conjugation strategy enabled successful generation and functional evaluation of the bioconjugate, alternative site-specific conjugation strategies like peptide–protein interactions may further improve product homogeneity, coupling efficiency and manufacturing reproducibility. Exploratory SEC purification indicated that removing residual protein species can increase the apparent purity of the bioconjugate. However, this improvement was offset by considerable loss of material. Notably, the comparable cAMP responses obtained with purified and non-purified C2IN-C3lim_E174Q_-Cys_His-AC suggested that residual protein species did not measurably compromise the functional activity of the bioconjugate in J774A.1 target cells within the limits of preliminary analysis. Nevertheless, optimizing chromatographic conditions or employing alternative purification strategies may be worthwhile in future studies, particularly when higher product purity and compositional homogeneity are required without compromising recovery. Moreover, the observed time-dependent loss of bioconjugate integrity is consistent with the known susceptibility of conventional maleimide-thiol linkages to deconjugation under physiological conditions [27]. The stronger decrease observed in PBS at 37 °C may additionally reflect changes in overall protein integrity, including degradation or aggregation. Importantly, under the FBS-containing conditions relevant for monocyte experiments performed in our study, substantial bioconjugate signal was retained within the experimentally relevant 24 h and 48 h periods. Nevertheless, further purification and the use of stabilized maleimide-based coupling strategies may, therefore, improve bioconjugate integrity in future applications.

Previous studies have shown that C3-based constructs preferentially target cells of the monocytic lineage, including macrophage-like cells, primary human monocytes, and osteoclasts, whereas substantially lower uptake has been observed in epithelial cells and fibroblasts [9,14,15,16]. In neutrophil-like cells, free C3 alone did not induce the characteristic morphological changes associated with intracellular C3 activity, suggesting inefficient functional delivery [28]. Consistent with these previous findings, the results of our study reflect the preferential targeting of monocytic cells described for C3-derived constructs [9,14,15,16]. However, in the present study, selectivity was experimentally assessed only against HeLa cells as a non-target cell model. Therefore, our findings demonstrate preferential targeting relative to HeLa cells rather than absolute selectivity for monocytic cells across different cell types. Future studies could further characterize the cell type selectivity of the bioconjugate in additional physiologically relevant target and non-target cell populations.

In addition, the intracellular trafficking of the bioconjugate remains incompletely understood. Although intracellular cAMP formation demonstrates successful delivery of catalytically active AC into a functional intracellular compartment, the precise uptake pathway, endosomal escape mechanism, intracellular stability, and cargo release remain objects of future investigations. Detailed intracellular trafficking studies will, therefore, be important to further optimize the delivery system and to better understand the underlying transport mechanism.

In addition, the downstream biological consequences of the AC-mediated cAMP elevation require more comprehensive investigation. The migration assays performed in the present study already demonstrated that intracellular delivery of AC is not only reflected by elevated intracellular cAMP levels but also translates into a measurable functional response of the target cells. The observed reduction in the proportion of migrating and chemotactic macrophages is consistent with the well-established role of cAMP signaling in regulating cytoskeletal dynamics and cell motility, indicating that the delivered AC remained biologically active following intracellular translocation [18,29]. Interestingly, treatment with unconjugated His-AC also resulted in a weaker, yet detectable, reduction in macrophage migration and chemotaxis. While the conjugated product showed substantially higher potency than His-AC alone in the migration assays, a minor contribution of unconjugated His-AC to the overall functional response cannot be completely excluded. Although no intracellular cAMP accumulation was detected, the modest reduction in macrophage migration observed upon His-AC treatment may reflect low-level membrane association or nonspecific cellular uptake. In this context, it has been reported that polyhistidine tags can enhance nonspecific interactions of recombinant proteins with negatively charged surfaces, suggesting that the N-terminal His-tag may have contributed to membrane association, although this remains speculative [30]. Such nonspecific interactions may, therefore, contribute to the modest functional effects observed for His-AC alone. While reduced macrophage migration demonstrates functional modulation of target cells, future studies should address the effects of intracellular AC delivery on inflammatory gene expression, cytokine production, macrophage polarization, differentiation of circulating monocytes into macrophages, phagocytic capacity, and antigen presentation. These are especially interesting questions since it has already been demonstrated that the complete CyaA toxin can influence macrophage function and differentiation in a cAMP-dependent manner [18]. Such investigations will be essential to define the full immunomodulatory potential of our approach and to assess whether the observed cellular effects translate into therapeutically meaningful outcomes in relevant disease models.

Beyond our proof-of-concept studies, potential therapeutic translation would require consideration of immunogenicity, biodistribution, systemic exposure, and off-target effects. Since non-target effects were evaluated only in HeLa cells in the present study, a broader assessment across physiologically relevant non-target cell populations will be required to define the selectivity and potential off-target profile of the approach. In particular, the bacterial origins of both C2IN-C3lim_E174Q_-Cys and His-AC may pose a risk of immunogenicity and could necessitate further optimization of their immunological and pharmacokinetic properties, for example, through protein engineering, PEGylation, or Fc domain fusion [31,32]. These aspects, however, represent longer-term considerations for the further development of the approach beyond the present academic proof-of-concept study.

## 4. Materials and Methods

***Molecular cloning and mutagenesis.*** Point mutations and insertions were carried out via inverse polymerase chain reaction (PCR) site-directed mutagenesis. As a template served the pGEX-2T plasmid containing the C2IN-C3lim sequence together with a glutathione-s-transferase (GST) tag and an ampicillin resistance gene. An additional C-terminal cysteine was introduced via overhang primers using the Q5^®^ Site-Directed Mutagenesis kit (New England Biolabs, Ipswich, MA, USA) according to the manufacturer’s instructions. Subsequently, the point mutation E174Q was introduced into the newly generated pGEX-2T:C2IN-C3lim-Cys plasmid via site-directed mutagenesis using mismatch primers with the Quick-Change XL Site-directed Mutagenesis Kit (Agilent Technologies, Santa Clara, CA, USA). After heat shock transformation, the generated plasmids were amplified in *Escherichia (E.) coli* DH5α (Clontech Laboratories, Inc., Heidelberg, Germany). Plasmids were purified according to the my-Budget plasmid mini kit (Bio-Budget Technologies GmbH, Krefeld, Germany) protocol. The correct sequence of the final pGEX-2T: C2IN-C3lim_E174Q_-Cys plasmid’s open reading frame was verified using Lightrun Sanger sequencing (Eurofins Genomics, Luxembourg, Luxembourg). The protein sequence of the adenylate cyclase (AC) domain of the CyaA toxin together with an N-terminal His-tag was introduced into a pET-21a(+) vector and synthesized by BioCat GmbH (Heidelberg, Germany).

***Recombinant protein expression of GST-C2IN-C3lim_E174Q_-Cys and His-AC.*** Heat shock transformation of chemical competent *Escherichia* (*E*.) *coli* BL21 (Novagene Madison, WI, USA) with the respective plasmid was performed. A single bacterial colony was transferred into 5 mL LB medium containing 100 μg/mL ampicillin and cultivated for 5 h at 37 °C with shaking at 180 rpm. The preculture was subsequently expanded overnight in 300 mL LB medium. For large-scale expression, 280 mL of the overnight culture were used to inoculate 8 L LB medium, which was incubated at 37 °C and 180 rpm until the culture reached an OD600 of 0.4–0.6. Protein production was induced by addition of 0.5 mM isopropyl-β-D-1-thiogalactopyranoside (IPTG, Carl Roth, Karlsruhe, Germany), followed by incubation at 16 °C (GST-C2IN-C3lim_E174Q_-Cys) or 29 °C (His-AC) for 20 h. Cells were harvested by centrifugation (5500× *g*, 10 min, 4 °C) and resuspended in 22 mL lysis buffer per liter main culture (10 mM NaCl, 20 mM Tris, pH 7.4) in case of GST-C2IN-C3lim_E174Q_-Cys or NPI-20 buffer (50 mM NaH_2_PO_4_, 300 mM NaCl, 20 mM imidazole, pH 8.0) in case of His-AC with addition of 1% Triton X-100 and 1% PMSF, respectively. Cell lysis was performed by using glass dounce homogenizer (Carl Roth, Karlsruhe, Germany) and EmulsiFlex-C3 homogenizer (Avestin Inc., Ottawa, ON, Canada) at 1000–1500 bar. After removal of insoluble material by centrifugation (13,000× *g*, 20 min, 4 °C), the supernatant was filtered through 0.45 μm and 0.2 μm syringe filters.

***Purification of recombinant GST-tagged C2IN-C3lim_E174Q_-Cys***. The clarified cell lysate was incubated overnight at 4 °C with Protino Glutathione Agarose 4B-beads (Macherey-Nagel, Dueren, Germany; 100 µL bed volume per liter main culture) preequilibrated in PBS pH 7.4. Following protein binding, the beads were washed twice with washing buffer (50 mM NaCl, 20 mM Tris-HCl; pH 7.4 and once with PBS pH 7.4. The GST-tagged recombinant protein was subsequently cleaved directly on the beads using 40 NIH units thrombin per liter of main culture (Amersham Biosciences, Little Chalfont, UK) for 1 h at room temperature. Meanwhile, 30 µL Benzamidine-Sepharose 6B beads (GE Healthcare, Chicago, IL, USA) per liter main culture were preequilibrated in PBS pH 7.4. After 1 h incubation, the Protino Glutathione Agarose 4B-beads were pelleted by centrifugation (10,000× *g*, 30 s, 4 °C), and the resulting supernatant was transferred to Benzamidine-Sepharose 6B beads and incubated for 10 min at room temperature to sequester residual thrombin. Following centrifugation (10,000× *g*, 30 s, 4 °C), the resulting supernatant was collected as the eluate. The elution procedure was repeated for a total of 10 cycles in PBS pH 7.4. Each eluate fraction was further treated with Benzamidine-Sepharose 6B beads under identical conditions to ensure efficient thrombin depletion. Protein concentration was determined by densitometric analysis of Coomassie-stained SDS-PAGE gels using a BSA standard curve for quantification. The purification resulted in a final yield of 9.09 mg of purified recombinant C2IN-C3lim_E174Q_-Cys from 8 L bacterial culture.

***Purification of recombinant His-AC***. His-AC was purified from filtered cell lysate via the His-Tag with an FPLC protocol at 4 °C. Therefore, an ÄKTA go protein purification system (Cytiva, Marlborough, MA, USA) was employed. Protino Ni-NTA FPLC columns with 1 mL column volume (CV) (MACHEREY-NAGEL, Dueren, Germany) were used with a flow rate of 1 mL/min for all steps. After an equilibration with 5 CV of NPI-20 buffer the cell lysate was loaded onto the column followed by washing with 18 CV of NPI-20 and an isocratic elution with 10 CV of NPI-250 buffer (50 mM NaH_2_PO_4_, 300 mM NaCl, and 250 mM imidazole, pH 8.0) in fractions of 0.5 mL each. Elution fractions were analyzed using SDS-PAGE and Coomassie staining with subsequent pooling of the fractions with high concentration and purity. For a buffer exchange to PBS dialysis (Spectra/Por^®^6, Spectrum Laboratories, Rockleigh, NJ, USA) with a dilution greater than 1:1000 was performed overnight at 4 °C. The protein concentration was determined against a BSA standard with densitometric analysis after SDS-PAGE with subsequent Coomassie staining revealing a final recombinant protein yield of 74.34 mg His-AC from 8 L bacterial culture.

***Chemical crosslinking of C2IN-C3lim_E174Q_-Cys and His-AC.*** For chemical crosslinking, C2IN-C3lim_E174Q_-Cys and His-AC were dialyzed in PBS conjugation buffer (pH 7.2); overnight at 4 °C prior use. His-AC was incubated with a five-fold molar excess of the heterobifunctional crosslinker m-maleimidobenzoyl-N-hydroxysuccinimide ester MBS (Thermo Fisher Scientific Inc., Waltham, MA, USA) for 2 h at 4 °C. Excess crosslinker was removed using Zeba™ Spin Desalting Columns (Thermo Fisher Scientific Inc., Waltham, MA, USA) according to the manufacturer’s instructions. Briefly, storage buffer was removed by centrifugation at 1500× *g* for 1 min, followed by equilibration with PBS (pH 7.2). Protein samples were applied to the columns and centrifuged for 2 min at 1500× *g* at 4 °C. Based on column recovery, an approximate 10% loss of protein was assumed after desalting. Desalted MBS-activated His-AC was subsequently mixed with C2IN-C3lim_E174Q_-Cys at a 1:1 molar ratio and incubated for 2 h at 4 °C to allow for conjugate formation. Chemical crosslinking was assessed by SDS-PAGE and Western blot under non-reducing conditions.

***Size exclusion chromatography of C2IN-C3lim_E174Q_-Cys_His-AC.*** Size exclusion chromatography was performed using a Superdex^®^ 200 Increase 10/300 GL column (Cytiva, Marlborough, MA, USA) in combination with an NGC™ Quest 10 Plus Chromatography System (Bio-Rad Laboratories, Inc., Hercules, CA, USA) and a flow rate of 0.5 mL/min. The column was first equilibrated with water and then with PBS conjugation buffer (pH 7.2). C2IN-C3lim_E174Q_-Cys_His-AC was applied via loop injections with consecutive runs of 500 µL. Elution of the protein was performed with 1.2 CV of PBS conjugation buffer (pH 7.2), and elution fractions were collected at 0.3 mL. Elution fractions were pooled according to the purity after SDS-PAGE and Coomassie staining and protein concentration was determined by densitometric analysis against a BSA standard. A total of 60 ng of bioconjugate was subjected to SEC, yielding approximately 20 ng of purified bioconjugate after size-exclusion chromatography.

***SDS-PAGE and Western blot.*** Protein samples were separated by sodium dodecyl sulfate polyacrylamide gel electrophoresis (SDS-PAGE) using 6% stacking gels and 12.5% running gels. Prior to electrophoresis, the samples were prepared in 5 × Laemmli sample buffer containing 0.3 M Tris-HCl, 10% SDS, 37.5% glycerol, 0.4 mM bromophenol blue, under non-reducing or reducing conditions (100 mM DTT), followed by heat denaturation at 95 °C for 10 min. Electrophoretic separation was carried out in SDS running buffer composed of 0.1% SDS, 27.2 mM Tris-HCl, and 192 mM glycine. Following gel electrophoresis, proteins were either stained with Coomassie Brilliant Blue R250 (SERVA Electrophoresis GmbH, Heidelberg, Germany) or transferred onto nitrocellulose membranes (Cytiva, Marlborough, MA, USA) by semi-dry blotting. Membranes were subsequently stained with Ponceau S solution (AppliChem GmbH, Darmstadt, Germany) to assess protein loading and transfer efficiency. Non-specific binding sites were blocked by incubation in 5% skim milk powder (Carl Roth, Karlsruhe, Germany) dissolved in PBS-T (PBS supplemented with 0.1% Tween-20) for 1 h at room temperature. To detect biotin-labeled proteins, the membrane was incubated with streptavidin peroxidase conjugate (1:5000 in PBS-T, Merck, Darmstadt, HE, Germany). Target proteins were identified by sequential incubation with primary and secondary antibodies either overnight at 4 °C or for 1 h at room temperature. The following antibodies were applied: anti-Hsp90 α/β antibody (F-8; 1:1000 in PBS-T; Santa Cruz Biotechnology, Dallas, TX, USA), anti-Adenylate Cyclase toxin [3D1] (1:500 in PBS-T; Absolute Antibody, Wilton, UK), anti-C2IN antiserum (rabbit, 1:10,000 in PBS-T), HRP-conjugated goat-anti-mouse IgG (H + L) secondary antibody (1:2500 in PBS-T; Thermo Fisher Scientific, Waltham, MA, USA), and HRP-conjugated goat-anti-rabbit IgG (H + L) secondary antibody (1:2500 in PBS-T; Thermo Fisher Scientific, Waltham, MA, USA). Horseradish peroxidase activity was visualized using a Pierce ECL Western Blotting Substrate (Thermo Fisher Scientific, Waltham, MA, USA). Chemiluminescence signals were detected either on medical X-ray films (AGFA Health Care, Mortsel, Belgium) in a darkroom or using the iBright™ CL1500 Imaging System (Thermo Fisher Scientific, Waltham, MA, USA).

***Protein concentration and chemical crosslinking efficiency determination.*** Protein concentrations were determined by densitometric analysis of Coomassie-stained SDS-PAGE gels using Fiji (ImageJ, version v1.52f; National Institutes of Health, Bethesda, MD, USA). Bovine serum albumin (BSA) standards of known concentration were included on the same gel and used for calibration. For each sample, the integrated band intensity (area under the curve, AUC) corresponding to the expected molecular weight of the protein of interest was quantified and compared with the respective BSA standards to determine the concentration of the target protein. Accordingly, protein concentrations reported and used throughout the experiments refer specifically to the concentration represented by the respective target protein band.

The apparent chemical crosslinking efficiency was determined analogously by densitometric analysis of Coomassie-stained SDS-PAGE gels. For each coupling reaction, the integrated intensities of all detectable protein bands within the corresponding lane were quantified. The AUC of the band corresponding to the expected molecular weight of the bioconjugate, C2IN-C3lim_E174Q_-Cys_His-AC, was divided by the cumulative AUC of C2IN-C3lim_E174Q_-Cys_His-AC, C2IN-C3lim_E174Q_-Cys, and His-AC protein bands in the respective lane. The apparent coupling efficiency was, thus, calculated as the fraction of the total densitometric signal corresponding to the coupling product.

***Bioconjugate integrity assay.*** The integrity of the bioconjugate C2IN-C3lim_E174Q_-Cys_His-AC was assessed over time under different conditions. The bioconjugate was diluted to a final concentration of 160 nM in PBS (pH 7.4), RPMI 1640 medium containing 1% penicillin/streptomycin, 5% heat-inactivated FBS, and 50 ng/mL M-CSF supplemented with either 5% or 10% heat-inactivated fetal bovine serum (FBS) and incubated at 37 °C in a humidified atmosphere containing 5% CO_2_. Samples corresponding to 80 ng of C2IN-C3lim_E174Q_-Cys_His-AC were collected after 0, 24, 48, and 72 h. Protein samples were mixed with Laemmli sample buffer and denatured under non-reducing conditions for 10 min at 95 °C at 1000 rpm. Samples were subsequently analyzed by SDS-PAGE followed by Western blotting. Membranes were probed with anti-C2IN antiserum (rabbit, 1:10,000 in PBS-T) and anti-Adenylate Cyclase toxin [3D1] (1:500 in PBS-T) to monitor the integrity of the respective bioconjugate components. To evaluate the stability of the bioconjugate under storage conditions, C2IN-C3lim_E174Q_-Cys_His-AC was additionally incubated in PBS (pH 7.4) at 4 °C for up to 72 h and samples were analyzed by SDS-PAGE followed by Coomassie staining to assess bioconjugate integrity over time. Integrated density values (IDVs) of the bioconjugate bands were determined by densitometric analysis. For each experimental condition, IDVs were normalized to the corresponding value at 0 h, which was set to 100%. The relative band intensity at subsequent time points was then expressed as a percentage of the respective 0 h value.

***Cell culture.*** Murine macrophage-like J774A.1 cells (DSMZ, Braunschweig, NI, Germany were cultured in DMEM medium (Gibco-Life Technologies, Carlsbad, CA, USA) supplemented with 10% fetal calf serum Gibco-Life Technologies, Carlsbad, CA, USA), 1% sodium pyruvate (Gibco-Life Technologies, Carlsbad, CA, USA), 1% MEM-NEAA (Gibco-Life Technologies, Carlsbad, CA, USA), and 100 U/mL (1%) penicillin–streptomycin (Gibco-Life Technologies, Carlsbad, CA, USA). HeLa (human cervix carcinoma cells; DSMZ, Braunschweig, Germany) cells were cultured in MEM medium (Gibco-Life Technologies, Carlsbad, CA, USA) supplemented with 10% fetal calf serum, 1% sodium pyruvate, 1% L-Glutamine (PAN-BIOTECH, Aidenbach, BY, Germany), 1% MEM-NEAA, and 100 U/mL (1%) penicillin–streptomycin. Both cell lines were sub-cultivated three times per week at 1:3 or 1:10. J774A.1 cells were detached by mechanical scraping, whereas HeLa cells were detached by trypsinization (PAN-BIOTECH, Aidenbach, BY, Germany).

***Isolation of primary human monocytes from buffy coats.*** Primary human monocytes were isolated from buffy coats of healthy human blood samples obtained from the Institute of Clinical Transfusion Medicine and Immunogenetics Ulm, Germany. The blood samples were provided anonymously within one day after donation, with no information available regarding sex, blood group, HLA type, or Rhesus factor. Human CD14^+^ monocytes were isolated from buffy coats by sequential density-gradient centrifugation and negative selection. Buffy coats were diluted with PBS pH 7.2 (Thermo Fisher Scientific, Waltham, MA, USA) and layered onto Ficoll–Paque density 1.077 g/mL (Cytiva, Marlborough, MA, USA). Following centrifugation at 700× *g* for 40 min without brake, the mononuclear cell layer was recovered from the plasma–Ficoll interface and washed with PBS pH 7.2 to remove residual Ficoll and platelets. To enrich monocytes within the mononuclear cell population, cells were resuspended in PBS pH 7.2 and OptiPrep density gradient medium (Serumwerk Bernburg, Bernburg, SA, Germany) was added. The cell suspension was subsequently subjected to a discontinuous density gradient consisting of Ficoll (1.077 g/mL), diluted Ficoll (1.068 g/mL), and a dextran-containing buffer layer (Merck, Darmstadt, HE, Germany). Following centrifugation at 600× *g* for 25 min, cells accumulating at the upper interphase, representing a monocyte-enriched fraction, were collected and washed. Purification of CD14^+^ monocytes was performed using the PluriSelect Monocyte Isolation System (PluriSelect Life Science, Leipzig, Germany) according to the manufacturer’s protocol. Briefly, the monocyte-enriched cell fraction was incubated with antibody-coated separation particles that selectively bind non-monocytic leukocyte populations. After incubation, the cell suspension was diluted with wash buffer (PluriSelect Life Science, Leipzig, Germany) and layered onto Ficoll (1.077 g/mL). Density-gradient centrifugation at 800× *g* for 15 min separated bead-bound cells from unbound monocytes. The CD14^+^ monocyte-enriched fraction was recovered from the density medium-plasma interface, washed, and resuspended in HEPES-buffered saline solution pH 7.0 (145 mM NaCl, 10 mM HEPES). Cell count and viability were determined by either trypan blue exclusion (Thermo Fisher Scientific, Waltham, MA, USA) or Calcein AM staining (Thermo Fisher Scientific, Waltham, MA, USA) using the Countess II FL automated cell counter system (Thermo Fisher Scientific, Waltham, MA, USA). Isolated CD14^+^ primary human monocytes were cultured for experiments on the following day in RPMI 1640 medium (PAN-Biotech, Aidenbach, BY, Germany) supplemented with 1% penicillin/streptomycin (Merck, Darmstadt, HE, Germany), 10% heat-inactivated fetal bovine serum (FBS; Cytiva, Marlborough, MA, USA), and 50 ng/mL M-CSF (Miltenyi Biotec, Bergisch Gladbach, NRW, Germany), or differentiated for 5 days in RPMI 1640 medium containing 1% penicillin/streptomycin, 5% heat-inactivated FBS, and 50 ng/mL M-CSF.

***Fluorescence microscopy.*** Either 1 × 10^4^ HeLa cells, 1.5 × 10^4^ J774A.1 cells or 3 × 10^4^ primary human monocytes were seeded per well into 8-well µ-slides (ibidi GmbH, Graefelfing, Germany) 24 h prior the experiment. For fluorescence microscopy analysis of monocyte-derived macrophages, 3 × 10^4^ primary human monocytes were seeded into 8-well µ-slides and differentiated as described above. The cells were treated with respective cell culture medium containing either C2IN-C3lim_E174Q_-Cys, His-AC or C2IN-C3lim_E174Q_-Cys_His-AC (160 nM each) for indicated time periods. Afterwards, cells were washed with washing buffer pH 7.4 (137 mM NaCl, 20 mM imidazole), fixed with 4% paraformaldehyde (diluted in PBS; Merck, Darmstadt, HE, Germany) for 20 min at room temperature followed by cell permeabilization using 0.4% Triton-X100 (diluted in PBS; Merck, Darmstadt, HE, Germany) for 5 min. Quenching was performed using 100 mM glycine (Merck, Darmstadt, HE, Germany) diluted in PBS for 2 min at room temperature. Cells were washed with PBS-T (PBS + 0.1% Tween-20; Merck, Darmstadt, HE, Germany) followed by blocking using 5% skimmed-milk powder (Merck, Darmstadt, HE, Germany) diluted in PBS-T for 30 min at 37 °C. Primary antibodies (anti-Adenylate Cyclase toxin [3D1] (1:100; Absolute Antibody, Wilton, UK), anti-C2IN antiserum (rabbit, 1:250)) diluted in 5% skimmed-milk powder in PBS-T were added for 1 h at room temperature. Following washing with PBS-T, cells were incubated with secondary antibodies (goat-anti-mouse IgG (H + L) AlexaFluor-568 (1:200; Thermo Fisher Scientific, Waltham, MA, USA), goat-anti-rabbit IgG (H + L) AlexaFluor-488 (1:500; Thermo Fisher Scientific, Waltham, MA, USA)) and SiR-actin (1:1000; Spirochrome AG, Stein am Rhein, Switzerland) diluted in 5% skimmed-milk powder in PBS-T for 1 h at room temperature. After washing with PBS-T, the nuclei were stained with Hoechst 33342 (1:5000 diluted in 5% skimmed-milk powder in PBS-T; Thermo Fisher Scientific, Waltham, MA, USA) for 5 min at room temperature. Cells were washed with PBS-T prior imaging in PBS with the Keyence BZ-X800 series fluorescence microscope (Keyence, Osaka, Japan). Regarding analyses of cellular structures and protein uptake, different filter cubes (actin: BZ-X filter Cy5 (OP-87766), nucleus: BZ-X filter DAPI (OP-87762), His-AC: BZ-X filter TRITC (OP-87764), C2IN-C3lim_E174Q_-Cys: BZ-X filter GFP (OP-87763)) were applied. For each cell type, fluorescence microscopy analyses were performed in two independent experiments.

***Cell morphology and cell viability assay.*** J774A.1 (3.8 × 10^3^ cells/well) or HeLa (1.5 × 10^3^ cells/well) cells were seeded in 96-well microtiter plates 24 h before treatment. For cell morphology assays the respective growth medium was replaced by fresh medium (negative control) or medium containing the proteins in indicated concentrations. After 48 h incubation time at 37 °C and 5% CO_2,_ cells were imaged using a Leica DMi1 microscope with a Leica MC170 HD camera (Leica, Wetzlar, Germany). As a positive control for cytotoxicity, cells were exposed to 40% dimethyl sulfoxide (DMSO) 1 h before cell viability measurement. Subsequently, the cell viability was assessed by adding 10% CellTiter 96^®^ AQueous One solution containing 3-(4,5-dimethylthiazol-2-yl)-5-(3-carboxymethoxyphenyl)-2-(4-sulfophenyl)-2H-tetrazolium, (MTS, Pro-mega, Madison, WI, USA). After an incubation for 1 h the absorbance at 492 nm was measured in a TriStar2 LB 942 microplate reader (Berthold Technologies GmbH & Co.KG, Bad Wildbad, Germany). Cell morphology and viability assays were performed in triplicates in three independent experiments.

**In vitro** ***enzyme activity of C3.*** To assess in vitro enzyme activity of C3 variants, HeLa cell lysate (30 µg total protein amount) was diluted in ADP-ribosylation buffer pH 7.5 (20 mM Tris-HCl, 1 mM EDTA, 1 mM DTT, 5 mM MgCl_2_, 1× cOmplete^TM^ protease inhibitor cocktail (Roche Diagnostics, Basel, Switzerland)) to a total volume of 20 µL per reaction. Simultaneously, 10 µM Biotin-NAD^+^ (Bio-Techne, Minneapolis, MN, USA) and C3 variants (64 nM) were added, followed by incubation for 30 min at 37 °C. The reactions were stopped by the addition of 5x Laemmli buffer containing DTT. After heating (95 °C, 10 min), SDS-PAGE and Western blot were conducted as described before. Western blot analysis and quantification were performed using Fiji (ImageJ; National Institutes of Health, Bethesda, MD, USA). The results were normalized to the respective loading and negative controls. In vitro enzyme activity analyses were performed in duplicates in four independent experiments. Statistical analysis was carried out as described below.

***Cyclic adenosine monophosphate (cAMP) enzyme-linked immunosorbent assay (ELISA).*** Changes in cAMP levels depending on C2IN-C3lim_E174Q_-Cys_His-AC in different cell types were assessed by enzyme-linked immunosorbent assays (ELISA). Therefore, primary human monocytes (5.8 × 10^5^ per well), J774A.1 and HeLa cells (1.5 × 10^5^ per well) were seeded in 48-well plates (Merck, Darmstadt, HE, Germany) 24 h prior ELISA. By means of monocyte-derived macrophages, 5.8 × 10^5^ primary human monocytes per well were seeded into 48-well plates and differentiated in the presence of 50 ng/mL M-CSF for 5 days prior use. Cells were preincubated with 3 µM Rolipram (Biomol, Hamburg, HH, Germany) for 1 h (37 °C, 5% CO_2_) followed by the addition of His-AC, C2IN-C3lim_E174Q_-Cys and C2IN-C3lim_E174Q_-Cys_His-AC in cell type-specific final concentrations (primary human monocytes: 80 nM, monocyte-derived macrophages: 120 nM, HeLa and J774A.1 cells: 40 nM). After incubation (1 h, 37 °C, 5% CO_2_), cells were washed with washing buffer pH 7.4 (137 mM NaCl, 20 mM imidazole) and PBS pH 7.4 followed by the incubation with AC inhibitors (20 µM adefovir and 50 µM calmidazolium chloride diluted in 137 mM NaCl pH 7.4). Cell lysis was performed by the addition of 0.1 M HCl + 0.4% Triton-X100 for 20 min (37 °C, 5% CO_2_). To assess cAMP levels in cell lysate, cAMP ELISA was performed according to manufacturer’s instructions (Enzo Biochem, Fermingdale, NY, USA). Optical density (OD) at 405 nm was measured using Tecan infinite M200 (Tecan, Maennedorf, Switzerland) plate reader. Blank-subtracted and non-specific binding-corrected OD values were used to determine cAMP concentrations from the standard curve. cAMP ELISAs were performed in duplicates in three independent experiments. Statistical analysis was carried out as described below.

***Monocyte motility assays.*** Following isolation, 1.5 × 10^4^ primary human monocytes were seeded into 18-well µ-slides (ibidi GmbH, Graefelfing, Germany) and treated with the indicated concentrations of His-AC or C2IN-C3lim_E174Q_-Cys_His-AC or left untreated (control). After 18 h of incubation, time-lapse images were recorded at one frame per minute over a 5 h period. Monocyte motility was analyzed using TrackMate (ImageJ, version 1.54). Briefly, cells were detected using the Laplacian-of-Gaussian (LoG) detector and linked over consecutive frames using the Kalman tracker. The tracking parameters were kept constant across all experiments. For subsequent analysis, the exported per-frame spot tables (track identity and x/y position) served as input. One dataset is integer-tracked (positions on the pixel grid). However, because integer quantization can distort very small displacements, we added uniform subpixel jitter of ±0.5 pixel before windowing. This jitter is immaterial to the net displacement of the window (typically 2–6 pixels), but it removes grid artifacts. Non-adherent cells carried by the medium flow were removed using a ballistic-gated directional filter. This filter deletes tracks that are straight and directionally coherent with the field’s net flow. It leaves confined, randomly oriented cells untouched. The filter was characterized using trajectory-level ground-truth simulations (sensitivity ≈ 0.90, specificity ≈ 0.998; production gate CR_(MIN) = 0.4, and isotropy threshold Z_(ISO) = 3). All model-free conclusions were confirmed to be unchanged with or without the filter. The remaining tracks were extracted and tiled into non-overlapping five-frame windows. These windows were then analyzed for standard movement quantities, such as net displacement, path length, confinement/straightness/directionality ratio, and speed. Net displacement (the straight-line distance from the starting point to the endpoint) over five consecutive frames served as a discriminator between motile and immobile cells. Over a short period, the net displacement of a directionally moving cell increases roughly proportionally to the number of frames, whereas the net displacement of a randomly wriggling cell increases only with the square root of that number.

***Dose–response analysis of monocyte motility.*** From the per-window net displacements described above, two fit-free readouts were computed per field of view (up to 800 windows per field were used, to limit within-track correlation). As a direct test of whether the bioconjugate slows cells rather than immobilizing them, the mean net displacement of the moving windows (those exceeding a threshold of 6 px) was taken as a per-window speed measure and compared across dose (migration-speed panel, Figure 7B). As a measure of how many cells were motile, the fraction of windows exceeding the 6 px threshold was computed. The threshold was set so that a stationary window essentially never reached it. Because a moving cell clears the threshold only about three times in four, this over-threshold′ fraction was rescaled per donor using the control-condition moving-cell scale to an absolute active-cell fraction. Since moving-cell speed was dose-invariant, this factor is dose-independent by setting the absolute level but leaving potency and slope unchanged. Fitting the uncorrected fraction (which needs no distributional assumption) gave the same conclusions. The active fraction was summarized against log_10_ dose with a falling four-parameter-logistic (4PL) curve, fitted as a nonlinear mixed-effects model (R package nlme, [33]) by maximum likelihood with the individual blood donor as the grouping factor. In Wilkinson-Rogers notation the complete model was active fraction~PL(log_10_ dose + treatment + 1|donor).

Here, the response is the per-field active-cell fraction, log_10_ dose is the concentration of the treatment substance, and treatment is a fixed two-level contrast (bioconjugate versus His-AC) and (1|donor) are donor random intercepts on the baseline and plateau only. The four logistic parameters are the low-dose baseline, the high-dose plateau, the log_10_ IC_50_ and the Hill slope (implicit in the Wilkinson notation and inherent to the 4PL model function). Random-effect structures were compared by AIC/BIC and screened for identifiability: donors differed in how many cells were motile (baseline, and marginally plateau) but not in potency or slope, so a donor-shared IC_50_ was used. Each treatment contrast was tested by its t-statistic and the overall toxin difference by a likelihood-ratio test against the treatment-pooled curve. The potency ratio and its confidence interval followed from the IC_50_ contrast. The Gaussian homoscedastic within-group error (the nlme default) was retained and checked by residual diagnostics. Analyses used R 4.5.2 with nlme 3.1-168. The R code was written with the assistance of Claude Code (Anthropic, version 2.1.191, Claude Opus 4.8, San Diego, CA, USA) and was assessed and tested by the authors.

***Macrophage chemotaxis.*** Following isolation, 1 × 10^5^ primary human monocytes were seeded into 8-well µ-slides (ibidi GmbH, Graefelfing, Germany) and incubated for 1 h in RPMI 1640 medium containing 1% P/S, 10% heat-inactivated FBS, and 50 ng/mL GM-CSF. Before the start of the experiment, the cells were treated with the indicated concentrations of His-AC or C2IN-C3lim_E174Q_-Cys_His-AC or were left untreated (control). Then, zymosan particles were added, and time-lapsed images were recorded at 1 frame/min over a 2 h period after another 20 min. Macrophage motility was analyzed using TrackMate (ImageJ; Supplementary Materials). In short, the cells were detected using a Laplacian-of-Gaussian (LoG) detector and linked across consecutive frames using a Kalman tracker. The tracking parameters remained constant throughout all experiments. Further analysis was performed using a custom Python script based on the Pandas, NumPy, and SciPy libraries. To ensure reliable tracking data, only trajectories containing at least 20 detected positions were included in the analysis. Non-adherent cells carried by the medium flow were again removed. To characterize the different migration behaviors, cell trajectories were classified into three populations based on their movement characteristics. We identified chemotactically directed migration by combining increased displacement (mean squared displacement (MSD) > 100 px^2^) with directional orientation toward the zymosan source (deviation angle ≤ 20°). Tracks showing increased movement without directional preference were classified as non-directed migration. Those remaining below the MSD threshold were classified as immobile or weakly mobile. The distribution of migration populations was calculated for each experimental condition and visualized graphically.

***Statistics.*** Unless stated otherwise, all experiments were independently performed at least three times. Individual biological replicates (*n*) are indicated for each experiment. Prior to statistical analysis, data were tested for normality using the Shapiro–Wilk test and for homogeneity of variances using Brown–Forsythe test. Normally distributed data with no significant heterogeneity of variances were assessed by assumptions of parametric testing, statistical significance was assessed by ordinary one-way ANOVA followed by Dunnett’s multiple comparisons test against the respective control group, as indicated. Statistical analyses were performed using GraphPad Prism version 10.6.1 (GraphPad Software, Boston, MA, USA). Probability values (*p*-values) were indicated as follows: not significant (ns), *p* ≥ 0.05; * *p* < 0.05; ** *p* < 0.005; *** *p* < 0.0005; **** *p* < 0.0001.

## 5. Conclusions

In conclusion, this study establishes proof-of-concept for the preferential targeting of monocytes and macrophages under the experimental conditions investigated here using the enzymatically inactive C3-toxin-derived transporter C2IN-C3lim_E174Q_-Cys conjugated to a biologically active protein cargo, while sparing non-target epithelial HeLa cells. The modular design of this approach separates cell targeting from cargo activity and thereby allows for different protein cargos to be evaluated using the same transporter without the need to generate new recombinant chimeric toxins. The induction of intracellular cAMP accumulation in murine and primary human target cells, together with the observed reduction in monocyte migration, demonstrates that the AC cargo remains functionally active following bioconjugate-mediated targeting. These findings highlight the potential of the platform as a flexible strategy for targeted protein delivery to monocytic cells. Future studies should further characterize the selectivity and biological effects of our bioconjugate in physiologically relevant non-target cells and disease-relevant models. These efforts will help establish how this platform can be harnessed to preferentially target and modulate monocyte- and macrophage-driven immune responses and may support its further development for targeted intervention in inflammatory lung diseases.

## Data Availability

The data presented in this study are available upon request from the corresponding authors.

## Supplementary Materials

The following supporting information can be downloaded at: https://www.mdpi.com/article/10.3390/ijms27188433/s1.

## Abbreviations

The following abbreviations are used in this manuscript:

AC	Adenylate cyclase
ADP	Adenosine diphosphate
ANOVA	Analysis of variance
ARDS	Acute respiratory distress syndrome
ATP	Adenosine triphosphate
*B. pertussis*	*Bordetella pertussis*
BSA	Bovine serum albumin
*C. botulinum*/*limosum*	*Clostridium botulinum*/*limosum*
cAMP	Cyclic adenosine monophosphate
C2	Binary *Clostridium botulinum* C2 toxin
C2I	Enzymatic component of the binary C2 toxin
C2II	Binding component of the binary C2 toxin
C2IIa	Proteolytically activated binding/translocation component of the binary C2 toxin
C2IN	N-terminal domain of C2I
C2IN-C3lim	Recombinant fusion protein consisting of C2IN fused to C3lim
C2IN-C3lim_E174Q_	Catalytically inactive C2IN-C3lim fusion protein carrying the E174Q substitution
C2IN-C3lim_E174Q_-Cys	C2IN-C3lim_E174Q_ containing an engineered C-terminal cysteine residue
C2IN-C3lim_E174Q_-Cys_His-AC	Chemically crosslinked bioconjugate consisting of C2IN-C3lim_E174Q_-Cys and His-tagged adenylate cyclase
C3	Clostridial C3 exoenzyme
C3bot	C3 exoenzyme from *Clostridium botulinum*
C3_E174Q_	Catalytically inactive C3 variant carrying the E174Q substitution
C3lim	C3 exoenzyme from *Clostridium limosum*
CD	Cluster of differentiation
CR3	Complement receptor 3 (CD11b/CD18)
CV	Column volume
CyaA	Adenylate cyclase toxin from *Bordetella pertussis*
DAPI	4’,6-Diamidino-2-phenylindole
DMEM	Dulbecco’s Modified Eagle Medium
DMSO	Dimethyl sulfoxide
DTT	Dithiothreitol
*E. coli*	*Escherichia coli*
E174Q	Glutamate-to-glutamine substitution at amino acid position 174
ECL	Enhanced chemiluminescence
EDTA	Ethylenediaminetetraacetic acid
ELISA	Enzyme-linked immunosorbent assay
FBS	Fetal bovine serum
Fc	Fragment crystallizable
FPLC	Fast protein liquid chromatography
GFP	Green fluorescent protein
GM-CSF	Granulocyte–macrophage colony-stimulating factor
GST	Glutathione S-transferase
GTP	Guanosine triphosphate
HeLa	Human cervical adenocarcinoma epithelial cell line
HEPES	4-(2-Hydroxyethyl)-1-piperazineethanesulfonic acid
His	Polyhistidine affinity tag
His-AC	Polyhistidine-tagged adenylate cyclase domain
HRP	Horseradish peroxidase
Hsp90	Heat shock protein 90
IC_50_	Half-maximal inhibitory concentration
IDVs	Integrated density values
IgG	Immunoglobulin G
IPTG	Isopropyl β-D-1-thiogalactopyranoside
J774A.1	Murine macrophage-like cell-line-derived from a BALB/c reticulum cell sarcoma
LB	Lysogeny broth
LoG	Laplacian of Gaussian
MAS	Macrophage activation syndrome
MBS	m-Maleimidobenzoyl-N-hydroxysuccinimide ester
MBS-His-AC	MBS-activated His-tagged adenylate cyclase
M-CSF	Macrophage colony-stimulating factor
MDM	Monocyte-derived macrophage
MEM	Minimum Essential Medium
MEM-NEAA	Minimum Essential Medium supplemented with non-essential amino acids
MSD	Mean squared displacement
MTS	3-(4,5-Dimethylthiazol-2-yl)-5-(3-carboxymethoxyphenyl)-2-(4-sulfophenyl)-2H-tetrazolium
NAD^+^	Nicotinamide adenine dinucleotide (oxidized form)
NC	Negative control
Ni-NTA	Nickel-nitrilotriacetic acid
NPI	Nickel purification imidazole buffer
OD	Optical density
PBS	Phosphate-buffered saline
PBS-T	Phosphate-buffered saline containing Tween 20
PCR	Polymerase chain reaction
PDE4	Phosphodiesterase 4
PEGylation	Polyethylene glycol conjugation
PHM	Primary human monocytes isolated from peripheral blood
PKA	Protein kinase A
PMN	Polymorphonuclear neutrophil
PMSF	Phenylmethylsulfonyl fluoride
Rho	Ras homolog family of small GTPases
RPMI	Roswell Park Memorial Institute medium
RTX	Repeats-in-toxin
SD	Standard deviation
SDS	Sodium dodecyl sulfate
SDS-PAGE	Sodium dodecyl sulfate-polyacrylamide gel electrophoresis
SEM	Standard error of the mean
SiR-actin	Silicon rhodamine-labeled actin probe
Tris	Tris(hydroxymethyl)aminomethane
TRITC	Tetramethylrhodamine isothiocyanate
