# Supplementary material for: Bioactive Conjugate Based on Bacterial Protein Toxins for Targeted Down-Modulation of Macrophage Functions
Source: Int J Mol Sci. 2026 Sep 21;27(18):8433. doi: 10.3390/ijms27188433 (PMC13607279; doi:10.3390/ijms27188433)
Supplement: Supplementary file 1 [file ijms-27-08433-s001.zip › IJMS_Manuscript_Revision2_Supplements.pdf]

Supplements:

A

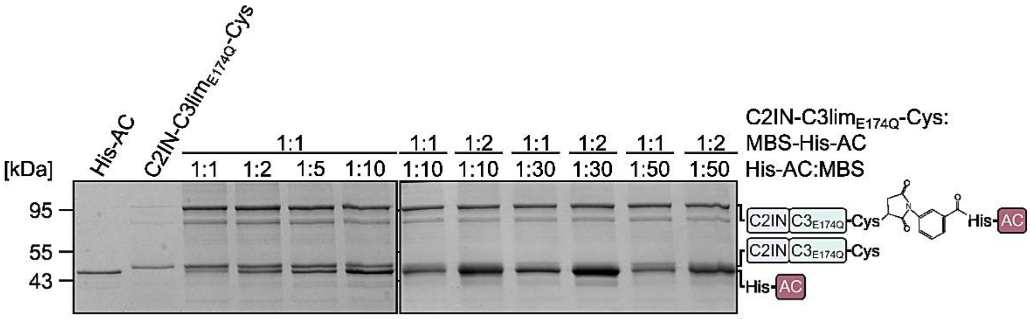

B

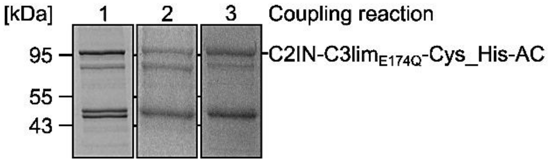

C

Coupling reaction	1	2	3
Coupling efficiency	35.3%	22.3%	36.3%
Mean ± SD	31.3 ± 7.8%		

Supplementary Figure S1: Characterization of the monocyte-targeting bioconjugate C2IN-C3lim$_{E174Q}$-Cys_His-AC formation. (**A**) Coomassie-stained SDS-PAGE showing the optimization of crosslinking ratios between His-AC to MBS and the MBS-coupled His-AC to the transporter domain C2IN-C3lim$_{E174Q}$-Cys. A final ratio of 1:5 (His-AC:MBS) and 1:1 (C2IN-C3lim$_{E174Q}$-Cys:MBS-His-AC) was used. (**B**) The reproducibility of the MBS-based coupling of C2IN-C3lim$_{E174Q}$-Cys_His-AC was assessed in three independent coupling reactions by Coomassie-stained SDS-PAGE. (**C**) Quantification of coupling efficiency and reproducibility of the three independent coupling reactions shown in (**B**). Coupling efficiency was determined by densitometric analysis of the corresponding band intensities. The three independent coupling reactions yielded a mean coupling efficiency of 31.3 ± 7.8% (n = 3, mean ± SD).

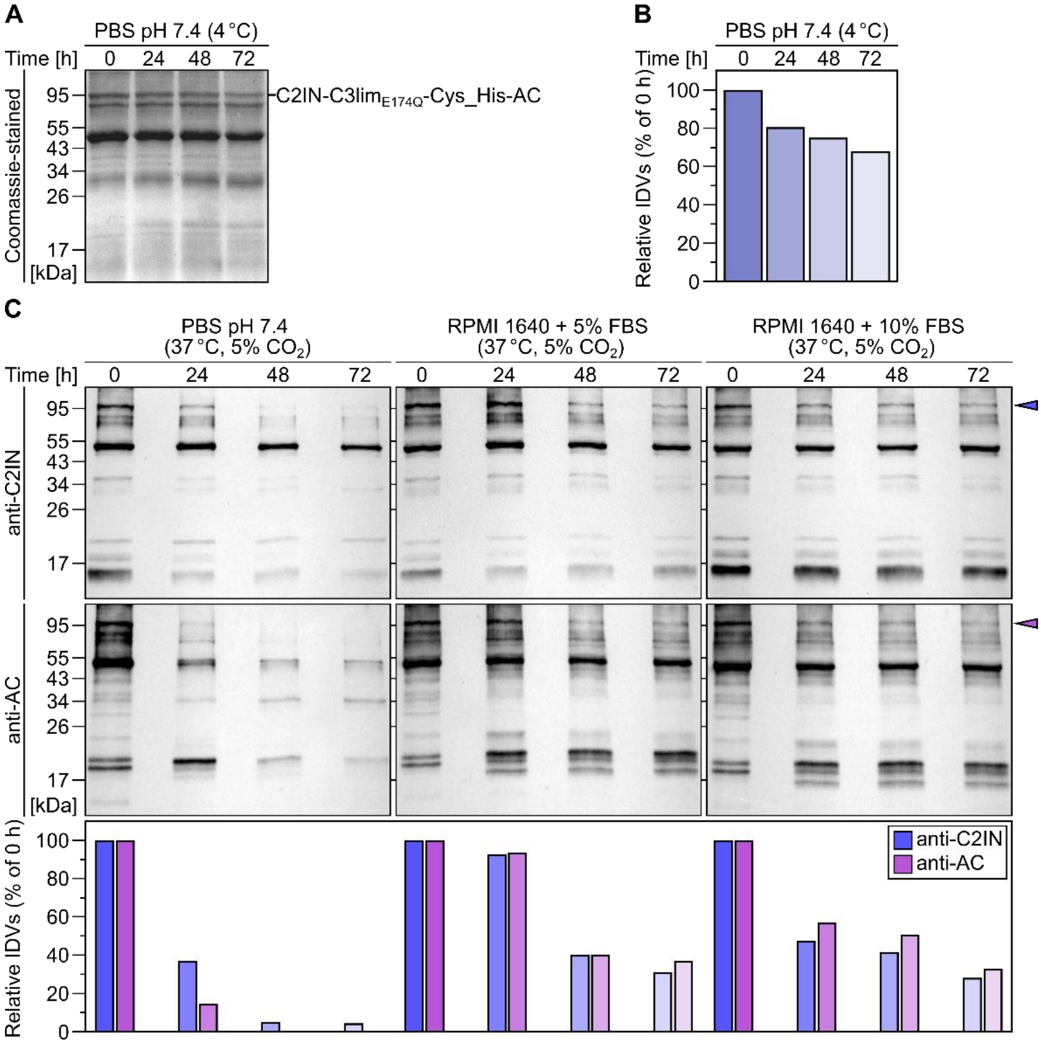

Supplementary Figure S2: Assessment of bioconjugate integrity under storage and cell culture conditions. **(A)** Bioconjugate integrity was assessed after incubation in PBS (pH 7.4) at 4 °C for up to 72 h by Coomassie-stained SDS-PAGE. **(B)** Densitometric quantification of the bioconjugate band shown in (**A**). Integrated density values (IDVs) were normalized to the signal at 0 h, set to 100%, and are expressed as relative IDVs (% of 0 h). **(C)** Bioconjugate integrity under cell culture conditions was evaluated at 37 °C in a humidified atmosphere containing 5% CO_2. Samples were incubated for up to 72 h in PBS or RPMI 1640 supplemented with 1% penicillin/streptomycin, 50 ng/mL M-CSF, and either 5% or 10% heat-inactivated FBS. Bioconjugate integrity was analyzed by Western blotting using antibodies against C2IN and AC. Bands corresponding to the bioconjugate are indicated by blue and purple arrowheads for anti-C2IN and anti-AC detection, respectively. Densitometric band intensities were normalized to the respective 0 h signal, which was set to 100%, and are presented relative to this baseline.

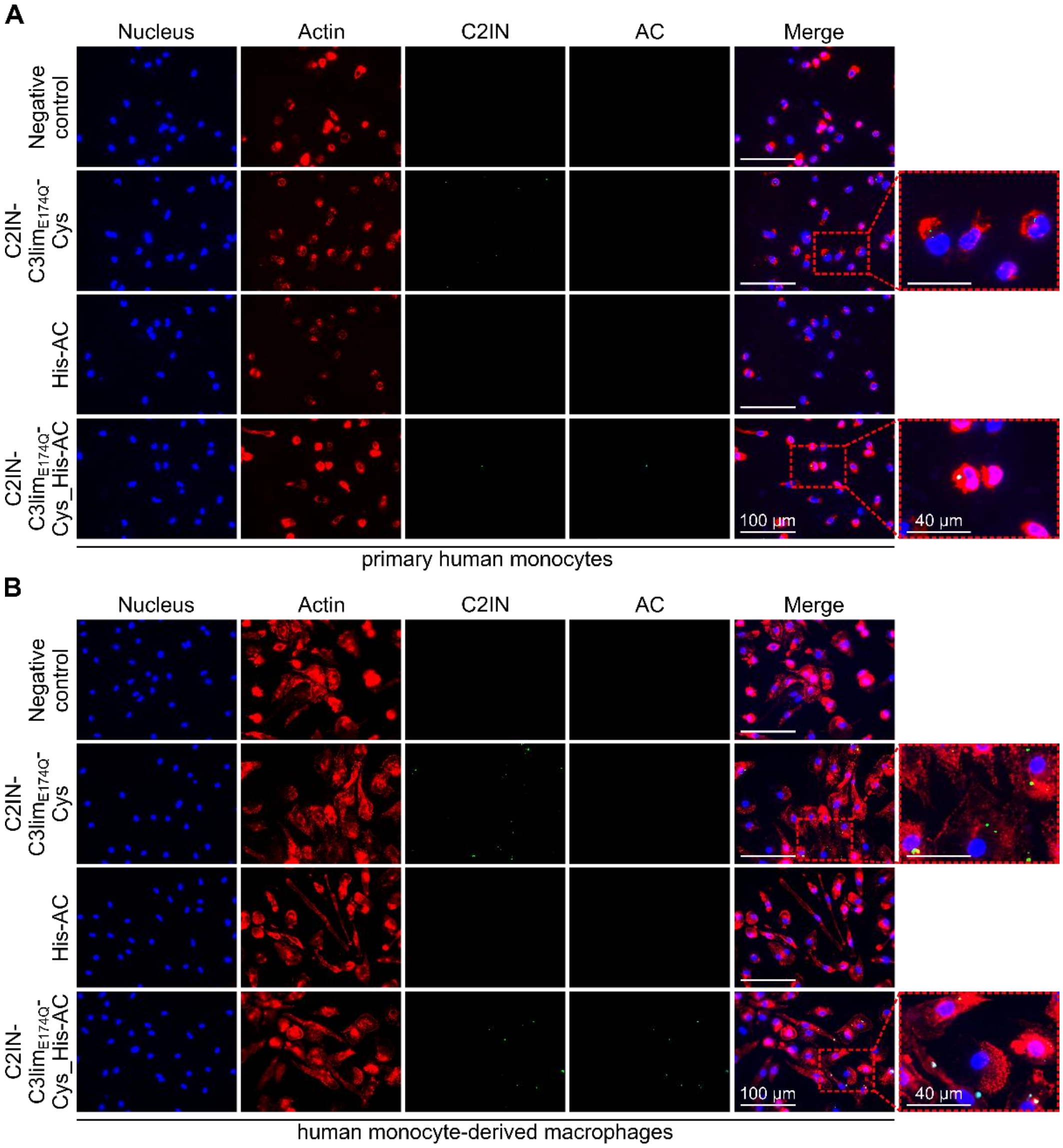

Supplementary Figure S3*:* Association of C2IN-C3lim$_{E174Q}$-Cys_His-AC with primary human monocytes and monocyte-derived macrophages. (**A**) Primary human monocytes isolated from human buffy coats were treated 20 h after isolation with 160 nM C2IN-C3lim$_{E174Q}$-Cys, His-AC or C2IN-C3lim$_{E174Q}$-Cys_His-AC for 4 h. Cells were then washed, fixed and immunostaining was performed using SiR-Actin (red, actin) and Hoechst (blue, nucleus). Cellular targeting by C2IN-C3lim$_{E174Q}$-Cys, His-AC or C2IN-C3lim$_{E174Q}$-Cys_His-AC was assessed by fluorescence microscopy using antibodies directed against C2IN (green) and AC (cyan). (**B**) Representative fluorescence microscopy images of monocyte-derived macrophages. Monocytes were isolated from human buffy coats and differentiated for 5 days in the presence of M-CSF. Treatment was performed on day 6 as described in (**A**). Scale bars are shown in representative images and correspond to 100 µm for all micrographs and 40 µm for all digital magnifications.

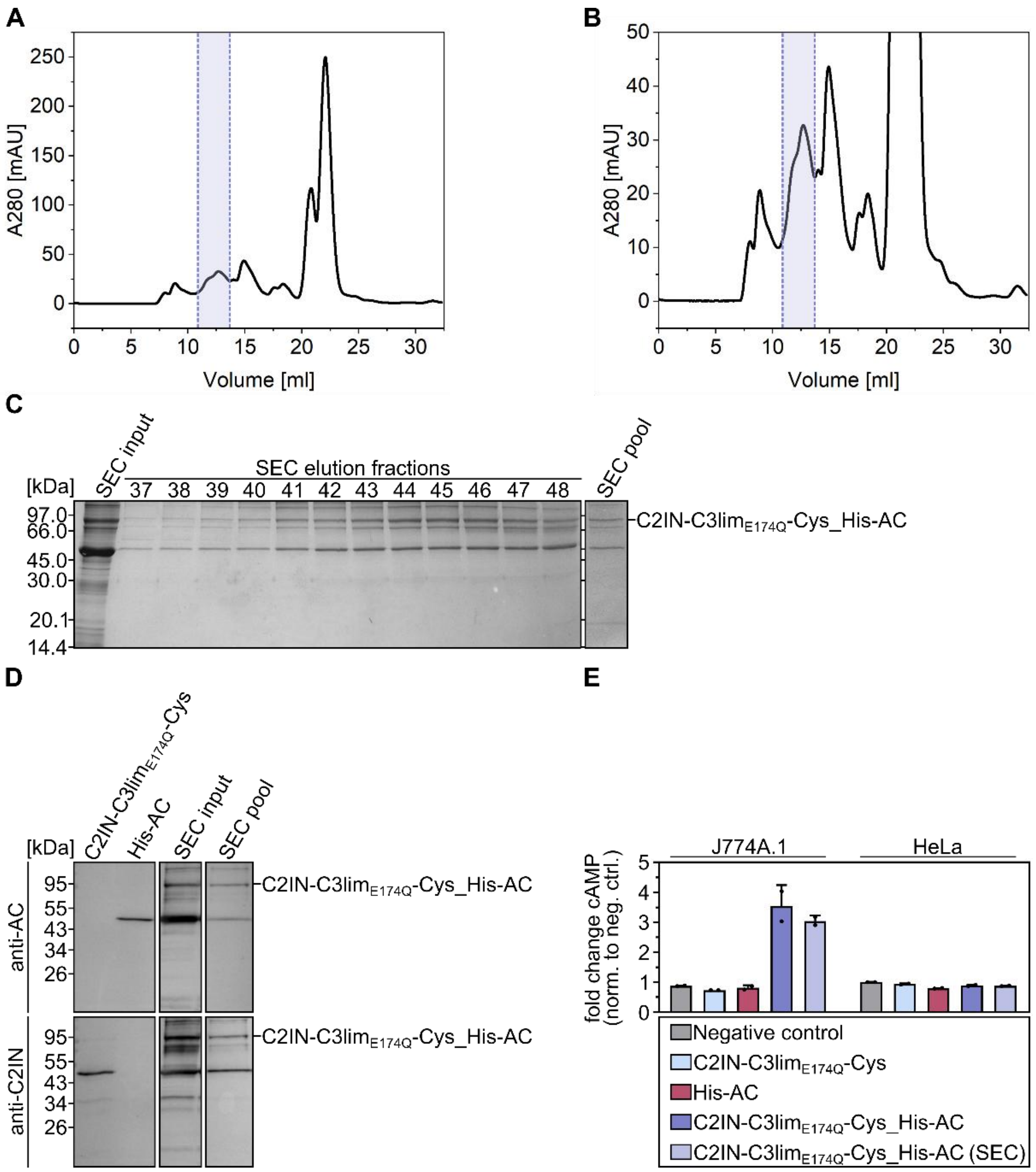

Supplementary Figure S4: Characterization of C2IN-C3lim$_{E174Q}$-Cys_His-AC after purification by size-exclusion chromatography. (**A**) Size-exclusion chromatogram of C2IN-C3lim$_{E174Q}$-Cys_His-AC displaying absorbance at 280 nm as a function of elution volume. The chromatographic region corresponding to the bioconjugate-enriched fractions is highlighted in light purple. (**B**) A magnification of the chromatographic region containing the bioconjugate-associated peak. (**C**) Coomassie-stained SDS-PAGE analysis of individual SEC elution fractions. Fractions 39-48 were combined to generate the SEC pool used for subsequent analyses. (**D**) Western blot analysis of C2IN-C3lim$_{E174Q}$-Cys, His-AC and C2IN-C3lim$_{E174Q}$-Cys_His-AC prior to SEC (SEC input) and following SEC purification and fraction pooling (SEC pool). Proteins were detected using antibodies directed against AC and C2IN. (**E**) Assessment of the functional activity of C2IN-C3lim$_{E174Q}$-Cys_His-AC before and after SEC purification, quantified by assessing intracellular cAMP levels in J774A.1 and HeLa cells. The cells were treated with C2IN-C3lim$_{E174Q}$-Cys, His-AC, non-SEC-purified or SEC-purified C2IN-C3lim$_{E174Q}$-Cys_His-AC, followed by examination of intracellular cAMP levels using a cAMP ELISA. Data are expressed as a fold change relative to the respective untreated control and represent one independent experiment performed with technical duplicates ($n = 1$).

Supplementary Movie S1: Time-lapse brightfield recording of macrophages and zymosan particles. Link:

Supplementary Movie S2: Time-lapse-tracking of individual cells from brightfield recordings. Link:
